# Comparing Direct TAVR to Balloon Aortic Valvuloplasty-TAVR in Patients with Cardiogenic Shock and Severe Aortic Stenosis—A TriNetX-Based Study

**DOI:** 10.3390/jcm15134943

**Published:** 2026-06-25

**Authors:** Aditya Desai, Simran Gill, Aparna Manoj, Naishal Mandal, Haidar Hajeh, Darshi Desai, Haresh Gandhi, Prabhdeep Sethi, James Blankenship, Tanawan Riangwiwat

**Affiliations:** 1 Department of Internal Medicine, University of California, Riverside School of Medicine, Riverside, CA 92521, USA; simran.gill@medsch.ucr.edu (S.G.); aparna.manoj@medsch.ucr.edu (A.M.); haidar.hajeh@medsch.ucr.edu (H.H.); darshi.desai@medsch.ucr.edu (D.D.); haresh.gandhi.md@gmail.com (H.G.);; 2 Department of Internal Medicine, Hurley Medical Center, Michigan State University of Human Medicine, Flint, MI 48824, USA; mandalnaishal01@gmail.com; 3 Division of Cardiology, University of New Mexico, Albuquerque, NM 87106, USA

**Keywords:** severe aortic stenosis, cardiogenic shock, TAVR, balloon aortic valvuloplasty, TriNetX analysis

## Abstract

**Objectives:** Severe aortic stenosis (AS) with cardiogenic shock (CS) presents a complex clinical challenge. For these patients, the optimal management strategy—either direct transcatheter aortic valve replacement (TAVR) or a staged approach with balloon aortic valvuloplasty (BAV) as a bridge to TAVR (BAV-TAVR)—is uncertain. We aimed to compare the outcomes of these two strategies. **Methods**: We conducted a retrospective cohort study using the TriNetX database. In this study, we identified patients with CS who underwent direct TAVR or who survived to and underwent TAVR within 30 days of balloon aortic valvuloplasty (BAV-TAVR). After matching propensity scores, 198 patients were analyzed in each group (total of 396). The primary outcome was major adverse cardiovascular events (MACE) at 30 days, 1 year, and 3 years. **Results**: The analysis included 396 matched patients (198 in each cohort). There was no significant difference in the primary endpoint of MACE at 30 days between the staged BAV and direct TAVR groups HR 1.14 (95% CI 0.79–1.64; *p* = 0.48), and this finding was consistent at 1 and 3 years with HR 1.20 (95% CI 0.89–1.61; *p* = 0.23) and HR 1.17 (95% CI 0.89–1.53; *p* = 0.25) respectively. Similarly, no differences were observed in secondary outcomes including all-cause mortality, stroke, and new permanent pacemaker implantation, at 30 days, 1, and 3 years. **Conclusions**: Among patients with severe AS and cardiogenic shock who survived definitive therapy, staged BAV-TAVR showed no detectable difference in short- or long-term outcomes versus direct TAVR. Given the limited sample size and the exclusion of patients who did not survive to TAVR, these results are hypothesis-generating and should not be read as evidence of equivalence or of bridging safety; prospective study is warranted.

## 1. Introduction

Aortic stenosis (AS) is the most common valvular disease in the developed world and carries high morbidity and mortality once symptomatic [1,2,3,4]. Without valve replacement, life expectancy is only 2–3 years [4]. Cardiogenic shock (CS), a severe complication of AS, is marked by low cardiac output and end-organ hypoperfusion, and remains associated with extremely high mortality [5,6].

Balloon aortic valvuloplasty (BAV) was historically used as palliative therapy due to its temporary hemodynamic benefit and poor long-term durability [7]. With the advent of TAVR, interest has grown in BAV as a stabilizing or bridging therapy. Current guidelines support BAV as a Class IIb option in critically ill patients, such as those with refractory pulmonary edema or CS, when immediate valve replacement is not feasible [8]. Contemporary studies suggest that BAV can be performed safely in these high-risk settings with careful attention to timing, as intervention within 48 h of shock onset is critical for the best outcomes [9,10,11,12].

The ideal timing of transcatheter interventions for individuals presenting with severe AS and concurrent cardiogenic shock remains poorly established. In contemporary clinical practice, two competing management strategies are frequently utilized. A direct-to-TAVR approach delivers definitive valve replacement in a single procedure but commits a hemodynamically unstable patient to definitive therapy before the shock state has been optimized. An alternative staged strategy utilizes emergent BAV to facilitate immediate hemodynamic stabilization, deferring TAVR until end-organ perfusion has been optimized. This bridging pathway may promote physiologic recovery and clarify prognosis while allowing for a more controlled index valve replacement, though it necessitates a second intervention and delays definitive therapy [9,10,11,12,13]. Which strategy yields better outcomes is unknown; the decision is mostly driven by operator judgment and institutional practice rather than comparative evidence. Randomized trials in this population are absent, and existing observational data are largely confined to small single-center series that lack a direct comparison between the two pathways and are underpowered for hard clinical endpoints [10,11,12]. This evidence gap is clinically consequential, as instances of cardiogenic shock complicating AS carry among the highest short-term mortality rates in structural heart disease [5,14].

As life expectancy increases, the burden of AS will inevitably rise. Given the poor prognosis for these patients and a lack of robust evidence-based guidelines, more research is required. This study aims to assess BAV’s role in bridging to TAVR (BAV-TAVR) compared to direct TAVR in patients with severe AS complicated by cardiogenic shock.

## 2. Methods

This retrospective cohort study utilized data from the TriNetX U.S. Collaborative Network, a federated cloud-based research platform comprising de-identified electronic health records from over 72 healthcare organizations across the United States. We identified adult patients diagnosed with severe aortic valve stenosis using ICD-10 codes I35.0 (nonrheumatic aortic valve stenosis), with concurrent cardiogenic shock (ICD-10 code R57.0), to form the eligible population for inclusion.

### Cohort Definition

Two mutually exclusive treatment cohorts were defined based on procedural codes.

Cohort 1 included patients who underwent TAVR alone, identified using CPT codes 33361 to 33369. Patients with prior surgical aortic valve replacement were excluded using CPT codes 33405, 33406 and 33410-33413, resulting in 1701 eligible individuals.

Cohort 2 consisted of patients who survived to and underwent TAVR as a separate procedure within 30 days of BAV (cohort entry was conditioned on the occurrence of TAVR; patients who underwent BAV but died or did not proceed to TAVR within this window were not captured—see Limitations). These individuals were identified using a combination of CPT codes 33361-33369 (TAVR) and CPT 92986 (BAV) and were similarly excluded if prior surgical valve replacement was recorded (CPT 33405, 33406 and 33410-33413), yielding 201 eligible patients.

To minimize confounding, we performed 1:1 propensity score matching between the two cohorts using a nearest-neighbor algorithm without replacement. Matching was based on multiple baseline characteristics including age, sex, race, comorbidities (e.g., ischemic heart disease, heart failure, atrial fibrillation, diabetes, hypertension, chronic kidney disease, cerebrovascular accident, cardiac arrest, malnutrition, or obesity/overweight), procedures (e.g., cardiac assist including impella and intra-aortic balloon pump (IABP), extracorporeal membrane oxygenation (ECMO)/extracorporeal life support (ECLS) services, and emergent endotracheal intubation), laboratory values (e.g., hemoglobin, creatinine, albumin, HbA1c, BMI, BNP, LVEF, and troponin) and medication (e.g., vasopressors). After matching, the final analytic sample comprised 396 patients: 198 patients who underwent direct TAVR alone and 198 who received BAV-TAVR. Outcomes were assessed over three predefined timeframes: 30 days, 1 year, and 3 years following the index procedure.

**Figure 1:**
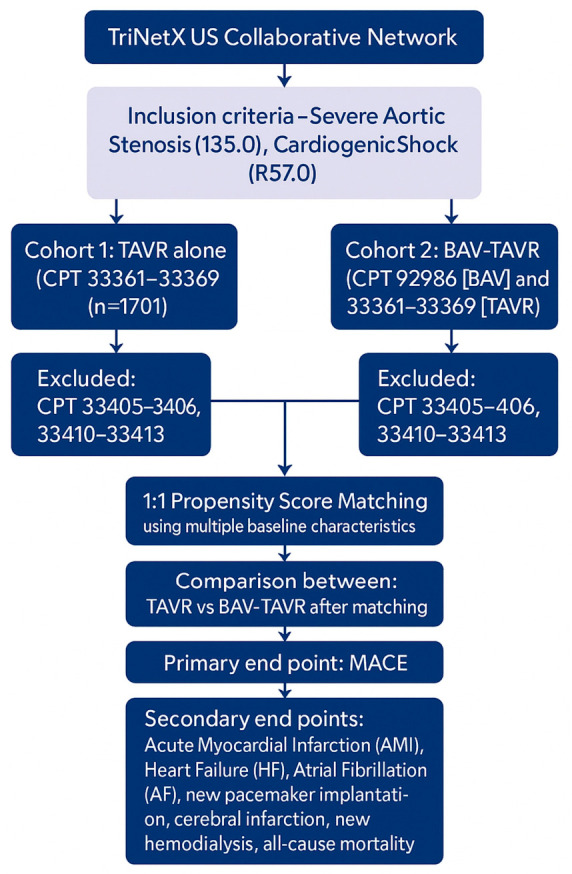
Schematic of study design using the TriNetX U.S. Collaborative Network. Legend: BAV = Balloon Aortic Valvuloplasty; TAVR = Transcatheter Aortic Valve Replacement; MACE = Major Adverse Cardiovascular Events.

The primary endpoint was the incidence of major adverse cardiovascular events (MACE), defined as a composite of all-cause mortality, acute myocardial infarction (AMI; ICD-10 I21.0–I21.4, I21.9 and ICD-9 410), and ischemic stroke/cerebral infarction (ICD-10 I63.0–I63.5, I63.8); all-cause mortality was ascertained from the deceased status flag and ICD-10 R99/R69. Repeat intervention (repeat valvuloplasty, repeat TAVR, or conversion to surgical aortic valve replacement) was not included in the composite. Secondary endpoints included individual incidence of acute myocardial infarction, heart failure, new pacemaker implantation, atrial fibrillation, cerebral infarction (stroke), new hemodialysis, and all-cause mortality. Figure 1 depicts the schematics of the study design.

A separate analysis of the treatment pathway was performed to determine the median time to TAVR which included patients from 40 HCOs and was not a part of this study. However, this was done to determine the median time to TAVR in patients who underwent BAV.

All statistical analyses were performed within the TriNetX platform on October 2nd, 2025, which ensures real-time access to de-identified, aggregated patient-level data while maintaining compliance with HIPAA and institutional ethical standards. Institutional Review Board approval was not required due to the de-identified nature of the database. Cox proportional hazard analysis was performed with TriNetX’s built-in software (https://live.trinetx.com/, accessed on 2 October 2025) to obtain the results, which were reported as hazard ratios; *p* < 0.05 was considered significant. Between-group comparisons of time-to-event outcomes were assessed using the log-rank test, the appropriate complement to the Kaplan–Meier hazard ratio, and the log-rank *p*-value is reported for each outcome. The proportional-hazards assumption was evaluated for every primary and secondary outcome at each timepoint using the platform’s built-in proportionality test; the assumption was not violated for any outcome (all *p* > 0.05).

To assess long-term clinical trajectories, Kaplan–Meier survival estimates for freedom from MACE and all-cause mortality were generated using the TriNetX platform. These estimates were plotted for propensity-matched cohorts across 1-year and 3-year follow-up intervals, incorporating 95% confidence intervals and between-group comparisons via the log-rank test. To ensure concise presentation, the 1-year (panel A) and 3-year (panel B) curves for each respective endpoint were consolidated into unified figures. Number-at-risk tables provided below each plot were calculated based on cohort sizes and survival function values at specified time intervals. For the all-cause mortality survival analysis, a small subset of patients (ten per cohort) was excluded due to outcomes occurring before the initiation of the follow-up window. Analysis file can be found in Supplementary Material. 

## 3. Results

### 3.1. Baseline Characteristics

Prior to propensity score matching, significant differences were observed between patients undergoing direct TAVR (*n* = 1701) and those receiving BAV-TAVR (*n* = 201). Patients in the BAV-TAVR group presented with a higher burden of comorbidities. Notably, the prevalence of heart failure was significantly greater in the BAV-TAVR cohort (97.5% vs. 80.7%; *p* < 0.001). Other cardiovascular diagnoses, including ischemic heart disease (87.1% vs. 77.8%; *p* = 0.002) and atrial fibrillation or flutter (61.2% vs. 50.4%; *p* = 0.004), were also significantly more common. Malnutrition was more frequently documented among BAV-TAVR patients (26.9% vs. 15%; *p* < 0.001), and they had significantly higher use of cardiac assist procedures (31.8% vs. 8.6%; *p* < 0.001), vasopressor use (44.4% vs. 81.1%; *p* < 0.001), emergency endotracheal intubation (14.4% vs. 5.4%; *p* < 0.001), and extracorporeal membrane oxygenation (5% vs. 1.5%; *p* = 0.001).

Laboratory profiles further highlighted the severity of illness in the BAV-TAVR cohort. Hemoglobin (9.7 ± 2.4 vs. 11.1 ± 2.3 g/dL; *p* < 0.001), serum albumin (3.1 ± 0.5 vs. 3.4 ± 0.6 g/dL; *p* < 0.001), and natriuretic peptide B levels (8134 ± 12,970 vs. 3788 ± 8156 pg/mL; *p* < 0.001) were significantly different between groups, suggesting greater hemodynamic compromise and malnutrition in the BAV-TAVR cohort. The median time to TAVR was 4 days from BAV.

Following 1:1 propensity score matching, 198 patients were included in each cohort. After matching, there were no statistically significant differences in baseline demographics, comorbidities, procedural characteristics, or laboratory values (all *p* > 0.05), indicating successful balancing of the cohorts for comparative analysis. Table 1 shows the patient characteristics before and after propensity matching, respectively.

### 3.2. Clinical Outcomes After 30 Days

At 30 days, there were no statistically significant differences between the direct TAVR and BAV-TAVR groups across all measured clinical outcomes. MACE, which was a composite of all-cause mortality, stroke and myocardial infarction, occurred in 30.3% of patients in the direct TAVR group compared with 27.3% in the BAV-TAVR group (HR: 1.14; 95% CI: 0.79–1.64; *p* = 0.48). All-cause mortality was observed in 15.3% of patients in the direct TAVR group and 13% of patients in the BAV-TAVR group (HR: 1.19; 95% CI: 0.70–2.04 *p* = 0.51).

**Figure 2:**
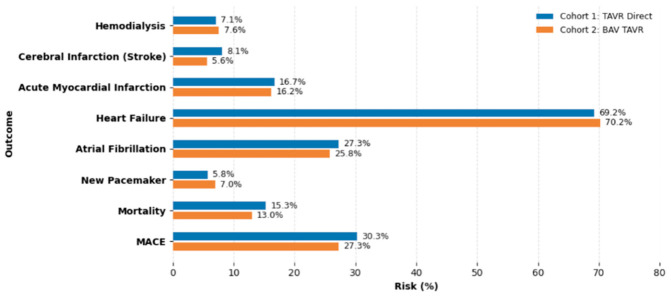
Risk percentages of clinical outcomes at 30 days after matching. MACE = composite of all-cause mortality, acute myocardial infarction, and ischemic stroke.

Other secondary outcomes were similarly balanced between groups. New permanent pacemaker implantation occurred in 5.8% of direct TAVR patients versus 7% in the BAV-TAVR group (*p* = 0.65). The incidence of atrial fibrillation was 27.3% in the direct TAVR group and 25.8% in the BAV-TAVR group (*p* = 0.65). Heart failure was reported to occur in 69.2% of the direct TAVR group compared with 70.2% of the BAV-TAVR group (*p* = 0.94). There were no significant differences in the rates of AMI (16.7% vs. 16.2%; *p* = 0.89), cerebral infarction (8.1% vs. 5.6%; *p* = 0.31), or new requirement for hemodialysis (7.1% vs. 7.6%; *p* = 0.83). The detailed results are shown in Table 2 and Figure 2.

### 3.3. Clinical Outcomes After 1 Year

At one-year follow-up, outcomes remained comparable between the two groups. MACE was observed in 47.5% of patients undergoing direct TAVR and 41.9% of those in the BAV-TAVR group (HR: 1.20; 95% CI: 0.89–1.61 *p* = 0.23). Mortality was 28.9% in the direct TAVR group compared to 22.8% in the BAV-TAVR group (HR: 1.32; 95% CI: 0.89–1.96; *p* = 0.16).

**Figure 3:**
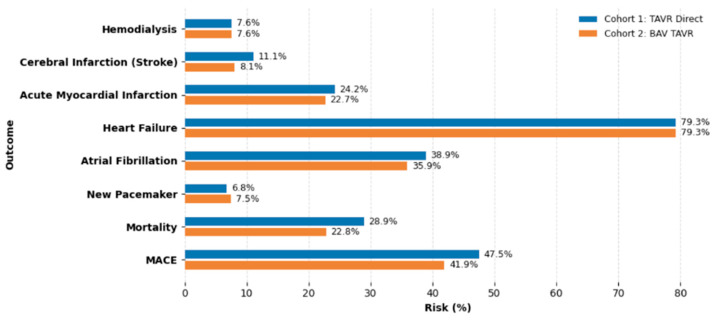
Risk percentages of clinical outcomes at 1 year after matching. MACE = composite of all-cause mortality, acute myocardial infarction, and ischemic stroke.

New permanent pacemaker implantation was similar in both groups (6.8% vs. 7.5%; *p* = 0.81), as was atrial fibrillation (38.9% vs. 35.9%; *p* = 0.45). The incidence of heart failure (79.3% in both; *p* = 0.67), AMI (24.2% vs. 22.7%; *p* = 0.64), cerebral infarction (11.1% vs. 8.1%; *p* = 0.26), and new hemodialysis (7.6% in both; *p* = 0.98) were also statistically indistinguishable. The detailed results are shown in Table 3 and Figure 3.

### 3.4. Clinical Outcomes After 3 Years

By three years post procedure, the long-term outcomes remained consistent with earlier findings. The cumulative incidence of MACE was 56.6% in the direct TAVR group and 52.5% in the BAV-TAVR group (HR: 1.17; 95% CI: 0.89–1.53; *p* = 0.25). Mortality was 39.5% for direct TAVR and 34.7% for BAV-TAVR (HR 1.21; 95% CI 0.87–1.69; *p* = 0.25), again showing no statistically significant difference.

**Figure 4:**
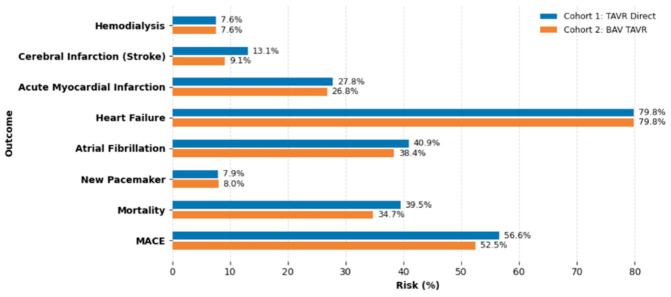
Risk percentages of clinical outcomes at 3 years after matching.

New permanent pacemaker implantation occurred in 7.9% of direct TAVR patients and 8.0% of BAV-TAVR patients (*p* = 0.98). Atrial fibrillation was reported in 40.9% and 38.4% of patients, respectively (*p* = 0.51). Heart failure was highly prevalent at three years, occurring in 79.8% of both direct TAVR and BAV-TAVR patients (*p* = 0.66). Rates of AMI (27.8% vs. 26.8%; *p* = 0.63), cerebral infarction (13.1% vs. 9.1%; *p* = 0.16), and hemodialysis (7.6% in both; *p* = 0.98) also remained statistically similar. The detailed results are shown in Table 4 and Figure 4.

**Figure 5:**
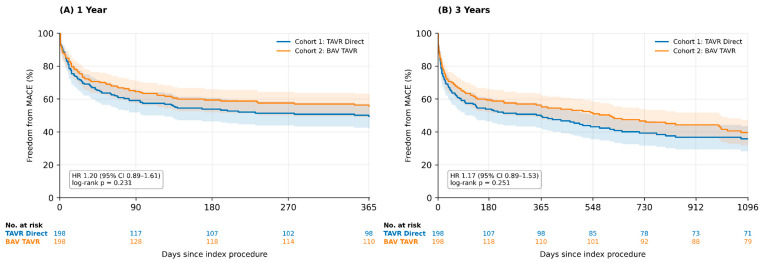
Kaplan–Meier estimates of freedom from MACE. (**A**) 1 year; (**B**) 3 years. Curves show propensity-matched cohorts (Cohort 1, direct TAVR; Cohort 2, BAV-TAVR); shaded bands are 95% confidence intervals, with the number-at-risk table below each panel. No patients were excluded for an outcome prior to the time window (at-risk at start: 198 and 198).

**Figure 6:**
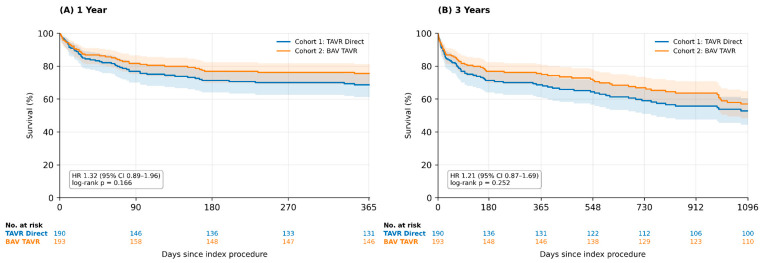
Kaplan–Meier estimates of mortality survival. (**A**) 1 year; (**B**) 3 years. Graphical representations indicate propensity-matched cohorts (Cohort 1, direct TAVR; Cohort 2, BAV-TAVR); shaded regions represent 95% confidence intervals, and the number-at-risk table is provided below each panel. Patients with outcomes occurring before the specified interval were excluded (at-risk at start: 190 and 193).

In addition, Kaplan–Meier estimates of freedom from MACE and of all-cause mortality survival are shown in Figure 5 and Figure 6, respectively, with 1-year results in panel A and 3-year results in panel B.

## 4. Discussion

### 4.1. TAVR vs. BAV-TAVR Outcomes

Our study concluded that in patients with severe AS complicated by CS, BAV-TAVR, in comparison to direct TAVR, does not compromise short- or long-term outcomes. These findings align with a large 2020 U.S. registry in which ~40% of patients underwent TAVR after bridging with BAV, typically within 90 days, and demonstrated comparable in-hospital and one-year outcomes to direct TAVR after propensity matching [13]. That study also reported lower index hospitalization costs in the BAV group. Therefore, BAV remains an important tool for their heart team in triaging, stabilizing, and optimizing patients when immediate TAVR is not yet feasible. The lack of a statistically significant result must be viewed as a failure to identify a disparity between the two cohorts, not as definitive proof of therapeutic parity.

Mechanistically, BAV temporarily relieves stenosis by enlarging the aortic valve area (AVA), lowering transvalvular gradients, and improving stroke volume and organ perfusion [12]. Improvement in renal function after BAV, an important prognostic marker in shock, has been documented following BAV and may explain why dialysis rates were not higher in our bridged group [14]. Hemodynamic improvement has been studied and confirmed these effects. Bularga et al. reported an average 8 mmHg reduction in mean gradient on echo at one week [15], while Kumar et al. demonstrated a 54% gradient reduction (40 → 18 mmHg) and AVA increase from 0.67 to 1.04 cm^2^ when measured invasively immediately post procedure [16]. Despite differences in measurement timing, both confirmed improved hemodynamics with acceptable procedural safety.

### 4.2. Role as a Bridge

BAV has mainly served as a temporizing measure in patients too unstable for valve replacement. Early studies demonstrated that patients selected for BAV typically had worse baseline status, yet still showed symptomatic and hemodynamic improvements comparable to direct TAVR once stabilized [10,11,17]. Later registry data reinforced that BAV is most beneficial as a bridge, since BAV alone carries high mortality [11]. A contrasting 2024 Japanese registry suggested that BAV–TAVR was associated with worse in-hospital outcomes compared to direct TAVR, though this likely reflects unadjusted baseline differences, as those that received BAV were older with more comorbidities [18].

### 4.3. Bridge-to-Decision and Diagnostic Value

Beyond serving as a bridge for stabilization and optimization, BAV can assist with determining patient eligibility. Saia et al. demonstrated that BAV can clarify candidacy when the heart team is uncertain, using BAV as a “bridge-to-decision,” as it helped to predict LV contractile reserve and mitral regurgitation grade reduction [19]. In low-LVEF patients, recovery was more likely when baseline gradients were higher (42 vs. 31 mmHg), and nearly all with EF improvement were subsequently selected for definitive valve replacement. Of note, the change in gradients, not AVA, were predictive of recovery.

### 4.4. Durability and Timing

Recent work has shown that BAV’s hemodynamic effects peak within 6 months, with improvements in EF (>40% at 1 month) predicting suitability for definitive replacement [20,21,22]. However, durability is limited, with a 2025 meta-analysis finding that delaying TAVR for staged BAV, on average 2 months to almost 1 year, was linked with higher mortality compared to immediate TAVR, although complication rates were similar [23]. These findings emphasize that BAV should be used selectively as a short-term bridge, ideally leading to definitive therapy as soon as possible. The median time in patients getting TAVR post BAV in CS was 4 days in the database.

### 4.5. Emerging Stepwise Strategies

There are emerging adjunctive strategies. A 2025 case report described a “BAV–PELLA–TAVR” approach, where BAV was combined with impella support as a stepwise bridge in three elderly patients with severe AS and shock, all of whom survived to definitive TAVR [24]. Similarly, Frerker et al. demonstrated how baseline cardiac output <3 L/min and renal impairment were strongly predictive of mortality after emergent TAVR, reinforcing the logic behind staged optimization with BAV and even mechanical circulatory support in selected patients [25].

### 4.6. Clinical Implications

Leveraging sophisticated low-profile technology alongside expanding expertise, a direct-to-TAVR strategy achieves prompt and enduring relief of transvalvular gradients, facilitating single-stage hemodynamic restoration while bypassing the added risks of vascular access, contrast load, and procedural delays inherent to bridging; consequently, this definitive pathway is often the favored primary intervention for many unstable individuals [26,27]. Consequently, BAV is an appropriate alternative as a temporizing bridge for distinct clinical scenarios, specifically when severe physiologic instability necessitates optimization prior to definitive replacement, or when a heart team faces diagnostic ambiguity regarding the valvular contribution to the shock state. Furthermore, its role remains critical for necessary hemodynamic evaluation or when anatomical and institutional factors prevent the immediate execution of TAVR [12,24].

### 4.7. Limitations

This study has several limitations inherent to retrospective analyses of federated administrative data. As a non-randomized cohort study relying on ICD-10/CPT codes to identify diagnoses, procedures, and outcomes without independent chart-level validation, which is not feasible within the de-identified platform, it is subject to misclassification and selection bias, and the cohort may not represent patients outside participating institutions. Administrative dating does not permit confirmation of the temporal sequence between shock onset and intervention, and prior BAV outside the study window was not captured. We also excluded patients who underwent BAV followed by TAVR during subsequent hospitalization beyond 30 days, and variability in coding and clinical practice across healthcare systems may limit generalizability. The retrospective design precludes causal inference, and hazard ratios were therefore used to summarize outcomes. Reassuringly, the high prevalence of vasopressor use (~81%) and mechanical circulatory support (~31%) in both matched cohorts is consistent with a genuinely shocked population, and these supportive therapies were included as matching covariates.

A central limitation is immortal time (survivorship) bias: because the BAV-TAVR cohort required survival to a TAVR occurring within 30 days of BAV, patients who underwent BAV but died or did not proceed to TAVR were not captured. This systematically favors the BAV-TAVR group by excluding early post-BAV mortality, so our findings apply only to patients who successfully bridged to TAVR and cannot be extrapolated to all patients undergoing BAV in cardiogenic shock. The federated platform’s aggregated output precluded a patient-level intention-to-treat or landmark analysis anchored at the time of BAV; the short median BAV-to-TAVR interval (4 days) limits but does not eliminate this bias. Because reported event rates are cumulative incidence estimates over each follow-up window, the design cannot attribute a given event to the bridging valvuloplasty versus the subsequent TAVR; isolating the procedure-specific hazard would require a patient-level analysis anchored separately at BAV and at TAVR, which the federated platform does not support. Furthermore, propensity matching balances only measured covariates, and unmeasured factors driving the choice of a staged approach hemodynamic instability, frailty, and valvular or vascular anatomy make residual confounding by indication likely. Finally, with 198 patients per group the study had approximately 80% power to detect only large differences in 30-day MACE (absolute risk difference ~13–14 percentage points); the observed differences were smaller, so a Type II error cannot be excluded.

## 5. Conclusions

In this propensity-score-matched analysis of patients with severe AS complicated by CS who successfully underwent transcatheter intervention, a staged BAV-TAVR strategy demonstrated comparable results to direct TAVR. No statistically significant differences in MACE or all-cause mortality were detected at the 30-day, 1-year, or 3-year follow-up intervals. These results should be considered hypothesis-generating due to the modest sample size and potential for Type II error. Importantly, our cohort definition required survival to the second procedure, meaning that these findings cannot be extrapolated to patients who fail to bridge from BAV to TAVR. Therefore, the lack of a detectable difference does not establish therapeutic equivalence or eliminate the risks associated with bridging. Our data suggest that BAV-TAVR remains a viable option for high-risk patients whose immediate candidacy for definitive therapy is uncertain, warranting further prospective studies to better define its role in clinical practice.

## Figures and Tables

**Table 1:** Baseline patient characteristics before and after propensity score matching.

Characteristic	Before Propensity Score Matching			After Propensity Score Matching		
	Direct TAVR (*n* = 1701)	BAV-TAVR (*n* = 201)	*p*-Value	Direct TAVR (*n* = 198)	BAV-TAVR (*n* = 198)	*p*-Value
**Demographics**						
Age at Index, mean ± SD	75.5 ± 10.6	76.4 ± 9.3	0.23	76.4 ± 9.8	76.3 ± 9.2	0.96
Male, *n* (%)	1003 (59.0)	112 (55.7)	0.37	114 (57.6)	112 (56.6)	0.83
White, *n* (%)	1347 (79.2)	145 (72.1)	0.02	144 (72.7)	144 (72.7)	1.00
Black or African American, *n* (%)	99 (5.8)	10 (5.0)	0.62	11 (5.6)	10 (5.1)	0.82
Asian, *n* (%)	46 (2.7)	11 (5.5)	0.02	10 (5.1)	11 (5.6)	0.82
**Diagnoses**						
Heart failure, *n* (%)	1372 (80.7)	196 (97.5)	<0.001	193 (97.5)	193 (97.5)	1.00
Ischemic heart diseases, *n* (%)	1323 (77.8)	175 (87.1)	0.002	172 (86.9)	172 (86.9)	1.00
Hypertensive diseases, *n* (%)	1344 (79.0)	171 (85.1)	0.04	167 (84.3)	169 (85.4)	0.77
Atrial fibrillation and flutter, *n* (%)	858 (50.4)	123 (61.2)	0.004	120 (60.6)	121 (61.1)	0.91
Chronic kidney disease, *n* (%)	803 (47.2)	108 (53.7)	0.08	106 (53.5)	107 (54.0)	0.92
Diabetes mellitus, *n* (%)	698 (41.0)	94 (46.8)	0.11	97 (49.0)	92 (46.5)	0.61
Malnutrition, *n* (%)	255 (15.0)	54 (26.9)	<0.001	53 (26.8)	53 (26.8)	1.00
Cerebrovascular diseases, *n* (%)	376 (22.1)	43 (21.4)	0.81	51 (25.8)	43 (21.7)	0.34
Overweight and obesity, *n* (%)	393 (23.1)	44 (21.9)	0.69	47 (23.7)	44 (22.2)	0.72
Cardiac arrest, *n* (%)	157 (9.2)	26 (12.9)	0.09	37 (18.7)	26 (13.1)	0.13
**Procedures**						
Impella and IABP, *n* (%)	147 (8.6)	64 (31.8)	<0.001	63 (31.8)	62 (31.3)	0.91
Emergency Endotracheal Intubation, *n* (%)	92 (5.4)	29 (14.4)	<0.001	26 (13.1)	28 (14.1)	0.77
ECMO or ECLS, *n* (%)	26 (1.5)	10 (5.0)	0.001	10 (5.1)	10 (5.1)	1.00
**Medications**						
Vasopressors, *n* (%)	756 (44.4)	163 (81.1)	<0.001	161 (81.3)	160 (80.8)	0.89
**Labs**						
Hemoglobin (g/dL), mean ± SD	11.1 ± 2.3	9.7 ± 2.4	<0.001	10.0 ± 2.5	9.8 ± 2.4	0.33
Creatinine (mg/dL), mean ± SD	1.6 ± 1.4	1.6 ± 1.5	0.91	1.7 ± 1.4	1.6 ± 1.5	0.95
Albumin (g/dL), mean ± SD	3.4 ± 0.6	3.1 ± 0.5	<0.001	3.1 ± 0.5	3.1 ± 0.5	0.68
BMI, mean ± SD	28.5 ± 6.7	28.1 ± 6.3	0.48	28.3 ± 6.5	28.2 ± 6.3	0.88
Natriuretic peptide B (pg/mL), mean ± SD	3788 ± 8,156	8134 ± 12,970	<0.001	7839 ± 11,747	7856 ± 13,029	0.99
LVEF (%), mean ± SD	37.2 ± 17.6	35.0 ± 17.5	0.35	32.7 ± 14.8	35.2 ± 17.7	0.39
Troponin I cardiac (ng/mL), mean ± SD	4.1 ± 25.1	5.2 ± 8.1	0.77	5.4 ± 9.7	5.2 ± 8.1	0.91

Data presented as *n* (%) or mean ± standard deviation (SD). TAVR: Transcatheter Aortic Valve Replacement; BAV: Balloon Aortic Valvuloplasty; IABP: Intra-aortic balloon pump; ECMO: Extracorporeal Membrane Oxygenation; ECLS: Extracorporeal Life Support; BMI: Body Mass Index; LVEF: Left Ventricular Ejection Fraction.

**Table 2:** Clinical outcomes at 30 days (after matching).

Outcome	Direct TAVR *n* (%)	BAV-TAVR *n* (%)	Hazard Ratio (95% CI)	Log-Rank *p*
**MACE**	60 (30.3)	54 (27.3)	1.14 (0.79–1.64)	0.485
**All-cause mortality**	29 (15.3)	25 (13.0)	1.19 (0.70–2.04)	0.518
**New pacemaker**	11 (5.8)	13 (7.0)	0.83 (0.37–1.86)	0.650
**Atrial fibrillation**	54 (27.3)	51 (25.8)	1.09 (0.75–1.60)	0.650
**Heart failure**	137 (69.2)	139 (70.2)	1.01 (0.80–1.28)	0.943
**Acute MI**	33 (16.7)	32 (16.2)	1.03 (0.63–1.68)	0.898
**Cerebral infarction (stroke)**	16 (8.1)	11 (5.6)	1.47 (0.68–3.18)	0.317
**Hemodialysis**	14 (7.1)	15 (7.6)	0.93 (0.45–1.92)	0.835

Data are presented as event counts with Kaplan–Meier estimated cumulative incidence (%). Because incidence is estimated accounting for censoring, percentages may not equal the event count divided by 198. Hazard ratios are from Cox proportional-hazards models; *p*-values are from the log-rank test. MACE = composite of all-cause mortality, acute myocardial infarction, and ischemic stroke. CI = confidence interval; MI = myocardial infarction.

**Table 3:** Clinical outcomes at 1 year (after matching).

Outcome	Direct TAVR *n* (%)	BAV-TAVR *n* (%)	Hazard Ratio (95% CI)	Log-Rank *p*
**MACE**	94 (47.5)	83 (41.9)	1.20 (0.89–1.61)	0.231
**All-cause mortality**	55 (28.9)	44 (22.8)	1.32 (0.89–1.96)	0.166
**New pacemaker**	13 (6.8)	14 (7.5)	0.91 (0.43–1.95)	0.815
**Atrial fibrillation**	77 (38.9)	71 (35.9)	1.13 (0.82–1.56)	0.459
**Heart failure**	157 (79.3)	157 (79.3)	1.05 (0.84–1.31)	0.675
**Acute MI**	48 (24.2)	45 (22.7)	1.10 (0.73–1.65)	0.645
**Cerebral infarction (stroke)**	22 (11.1)	16 (8.1)	1.44 (0.75–2.73)	0.268
**Hemodialysis**	15 (7.6)	15 (7.6)	1.00 (0.49–2.04)	0.989

Data are presented as event counts with Kaplan–Meier estimated cumulative incidence (%). Because incidence is estimated accounting for censoring, percentages may not equal the event count divided by 198. Hazard ratios are from Cox proportional-hazards models; *p*-values are from the log-rank test. MACE = composite of all-cause mortality, acute myocardial infarction, and ischemic stroke. CI = confidence interval; MI = myocardial infarction.

**Table 4:** Clinical outcomes at 3 years (after matching).

Outcome	Direct TAVR *n* (%)	BAV-TAVR *n* (%)	Hazard Ratio (95% CI)	Log-Rank *p*
**MACE**	112 (56.6)	104 (52.5)	1.17 (0.89–1.53)	0.251
**All-cause mortality**	75 (39.5)	67 (34.7)	1.21 (0.87–1.69)	0.252
**New pacemaker**	15 (7.9)	15 (8.0)	0.99 (0.49–2.03)	0.986
**Atrial fibrillation**	81 (40.9)	76 (38.4)	1.11 (0.81–1.52)	0.514
**Heart failure**	158 (79.8)	158 (79.8)	1.05 (0.84–1.31)	0.661
**Acute MI**	55 (27.8)	53 (26.8)	1.09 (0.75–1.60)	0.636
**Cerebral infarction (stroke)**	26 (13.1)	18 (9.1)	1.53 (0.84–2.79)	0.163
**Hemodialysis**	15 (7.6)	15 (7.6)	1.00 (0.49–2.04)	0.989

Data are presented as event counts with Kaplan–Meier estimated cumulative incidence (%). Because incidence is estimated accounting for censoring, percentages may not equal the event count divided by 198. Hazard ratios are from Cox proportional-hazards models; *p*-values are from the log-rank test. MACE = composite of all-cause mortality, acute myocardial infarction, and ischemic stroke. CI = confidence interval; MI = myocardial infarction.

## Data Availability

The data supporting the findings of this study are derived from the TriNetX platform. As TriNetX provides access to de-identified, aggregate patient-level data subject to license agreements, the data are not publicly available but can be accessed through the TriNetX platform with appropriate institutional authorization.

## Supplementary Materials

The following supporting information can be downloaded at: https://www.mdpi.com/article/10.3390/jcm15134943/s1, TriNetX analysis file.

## Abbreviations

The following abbreviations are used in this manuscript: AS, aortic stenosis; CS, cardiogenic shock; TAVR, transcatheter aortic valve replacement; BAV, balloon aortic valvuloplasty; MACE, major adverse cardiovascular events; AMI, acute myocardial infarction; HR, hazard ratio; CI, confidence interval; IABP, intra-aortic balloon pump; ECMO, extracorporeal membrane oxygenation; ECLS, extracorporeal life support; LVEF, left ventricular ejection fraction; BNP, B-type natriuretic peptide; BMI, body mass index; ICD, International Classification of Diseases; CPT, Current Procedural Terminology; IRB, Institutional Review Board.
