# Supplementary material for: Comparing Direct TAVR to Balloon Aortic Valvuloplasty-TAVR in Patients with Cardiogenic Shock and Severe Aortic Stenosis—A TriNetX-Based Study
Source: J Clin Med. 2026 Jun 25;15(13):4943. doi: 10.3390/jcm15134943 (PMC13361323; doi:10.3390/jcm15134943)
Supplement: Supplementary file 1 [file jcm-15-04943-s001.zip › jcm-4369480-supplementary.pdf]

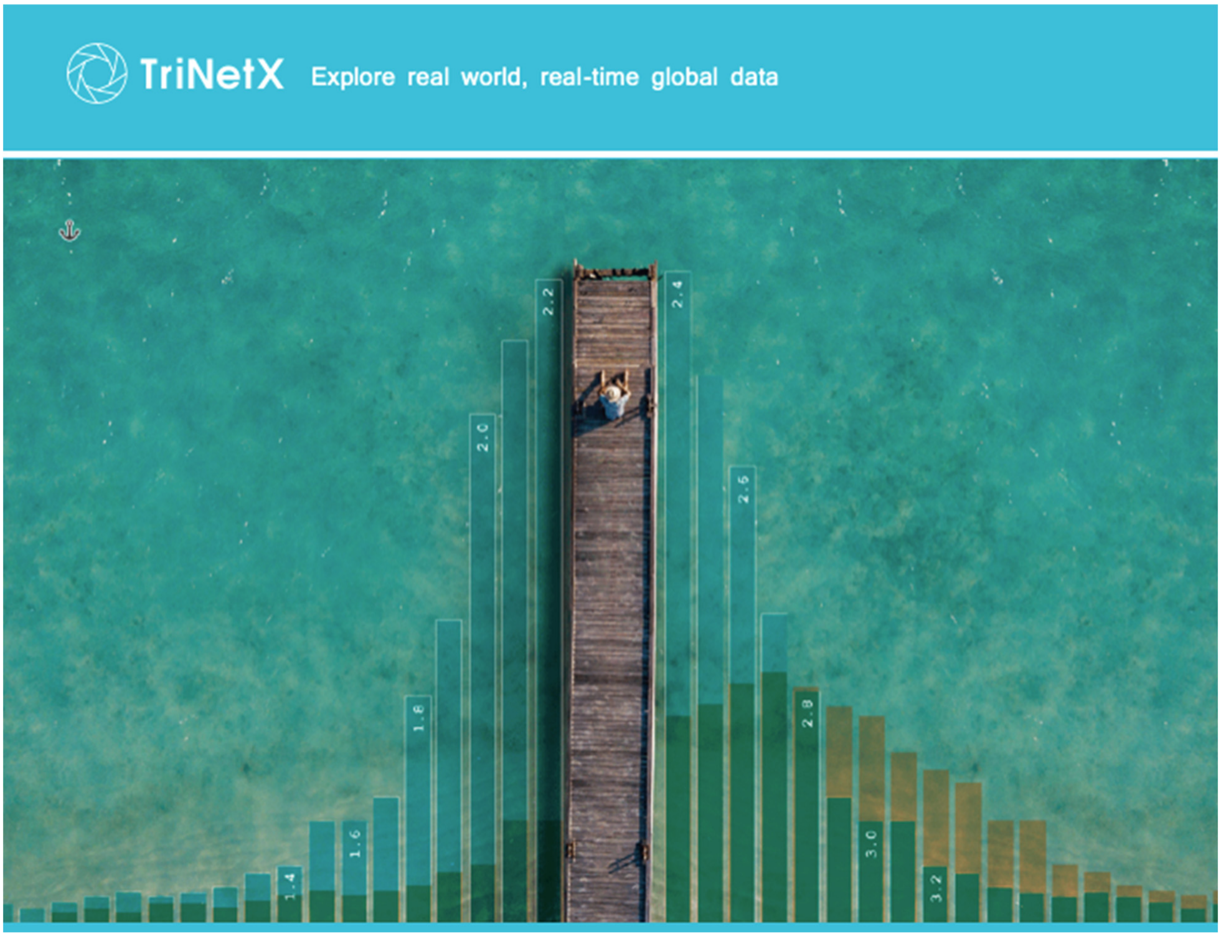

AS CS v. valvuloplasty

Compare Outcomes Analysis

Created by TriNetX on Oct 3, 2025, 04:44:24 UTC

Introduction

TriNetX is the global federated health research network providing access to electronic medical records (diagnoses, procedures, medications, laboratory values, genomic information) across large healthcare organizations (HCOs). This report was run on the set of HCOs grouped into a network called US Collaborative Network. This network included 69 HCO(s).

This report describes a Compare Outcomes Analysis, named Direct TAVR-BAV-TAVR 30 mace days (1) (1), generated by the TriNetX platform on Oct 3, 2025, 04:44:24 UTC. This analysis compared the outcomes of two cohorts: Cohort A (1,581 patients) named TAVR Direct Final and Cohort B (187 patients) named BAV TAVR Final.

This analysis was run by Aditya Desai (adityad@medsch.ucr.edu) and downloaded by Aditya Desai (adityad@medsch.ucr.edu).

Methods

The analysis process includes two main steps: 1) Defining the cohorts through query criteria; 2) Setting up and running the analysis. Setting up the analysis requires definitions for the index event, outcomes criteria, and the time frame. Compare outcomes supports four analyses: Measures of Association, Survival, Number of Instances and Lab result distribution. These analyses have additional options that are listed in the Outcomes Definitions and Analyses Specifications section below. Furthermore, characteristics of the cohorts that are balanced using propensity score matching are also included in the Propensity Score Matching section.

Cohorts definition

This section lists all terms used in the definitions of the two cohorts.

Query Criteria for Cohort 1 (query name: TAVR Direct Final)

This query was run on the network US Collaborative Network with 69 HCO(s) queried and 69 HCO(s) responded. A total of 43 provider(s) responded with patients. The final cohort included 1,581 patients who matched the query criteria listed in the table below. For the text representation of the query criteria please see Appendix A.

**Group 1**					
	**Group 1A**				
	must have		diagnosis	UMLS:ICD10CM:I35.0	Nonrheumatic aortic (valve) stenosis
		and	diagnosis	UMLS:ICD10CM:R57.0	Cardiogenic shock
	cannot have		procedure	UMLS:CPT:33410	Replacement, aortic valve, open, with cardiopulmonary bypass; with stentless tissue valve
		or	procedure	UMLS:CPT:33405	Replacement, aortic valve, open, with cardiopulmonary bypass; with prosthetic valve other than homograft or stentless valve
		or	procedure	UMLS:ICD10PCS:02RF0JZ	Replacement of Aortic Valve with Synthetic Substitute, Open Approach

	or	procedure	UMLS:CPT:33405	Replacement, aortic valve, open, with cardiopulmonary bypass; with prosthetic valve other than homograft or stentless valve
	or	procedure	UMLS:CPT:33411	Replacement, aortic valve; with aortic annulus enlargement, noncoronary sinus
	or	procedure	UMLS:ICD10PCS:02RF08Z	Replacement of Aortic Valve with Zooplastic Tissue, Open Approach
	or	procedure	UMLS:ICD10PCS:02RF0KZ	Replacement of Aortic Valve with Nonautologous Tissue Substitute, Open Approach
	or	procedure	UMLS:ICD10PCS:02RF07Z	Replacement of Aortic Valve with Autologous Tissue Substitute, Open Approach
	or	procedure	UMLS:CPT:33413	Replacement, aortic valve; by translocation of autologous pulmonary valve with allograft replacement of pulmonary valve (Ross procedure)
date constraint		The terms in this group occurred at any time		
event relationship		Any instance of Group 1B occurred within 10 days on or after any instance of Group 1A		
**Group 1B**				
must have	any of	procedure	UMLS:CPT:33363	Transcatheter aortic valve replacement (TAVR/TAVI) with prosthetic valve; open axillary artery approach
		procedure	UMLS:CPT:33362	Transcatheter aortic valve replacement (TAVR/TAVI) with prosthetic valve; open femoral artery approach
		procedure	UMLS:CPT:33361	Transcatheter aortic valve replacement (TAVR/TAVI) with prosthetic valve; percutaneous femoral artery approach
		procedure	UMLS:CPT:33364	Transcatheter aortic valve replacement (TAVR/TAVI) with prosthetic valve; open iliac artery approach
		procedure	UMLS:SNOMED:773996000	Transcatheter aortic valve implantation
		procedure	UMLS:CPT:1021150	Transcatheter aortic valve replacement (TAVR/TAVI) with prosthetic valve
		procedure	UMLS:CPT:33368	Transcatheter aortic valve replacement (TAVR/TAVI) with prosthetic valve; cardiopulmonary bypass support with open peripheral arterial and venous cannulation (eg, femoral, iliac, axillary vessels) (List separately in addition to code for primary procedure)
		procedure	UMLS:CPT:33369	Transcatheter aortic valve replacement (TAVR/TAVI) with prosthetic valve; cardiopulmonary bypass support with central arterial and venous cannulation (eg, aorta, right atrium, pulmonary artery) (List separately in addition to code for primary procedure)
		procedure	UMLS:CPT:33367	Transcatheter aortic valve replacement (TAVR/TAVI) with prosthetic valve; cardiopulmonary bypass support with percutaneous peripheral arterial and venous cannulation (eg, femoral

				vessels) (List separately in addition to code for primary procedure)

Query Criteria for Cohort 2 (query name: BAV TAVR Final)

This query was run on the network US Collaborative Network with 72 HCO(s) queried and 70 HCO(s) responded. A total of 32 provider(s) responded with patients. The final cohort included 187 patients who matched the query criteria listed in the table below.

**Group 1**				
**Group 1A**				
must have		diagnosis	UMLS:ICD10CM:I35.0	Nonrheumatic aortic (valve) stenosis
	and	diagnosis	UMLS:ICD10CM:R57.0	Cardiogenic shock
	and any of	procedure	UMLS:CPT:92986	Percutaneous balloon valvuloplasty; aortic valve
		procedure	UMLS:ICD10PCS:027F3ZZ	Dilation of Aortic Valve, Percutaneous Approach
		procedure	UMLS:CPT:1012998	Percutaneous balloon valvuloplasty
		procedure	UMLS:ICD10PCS:027F4ZZ	Dilation of Aortic Valve, Percutaneous Endoscopic Approach
		procedure	UMLS:CPT:92986	Percutaneous balloon valvuloplasty; aortic valve
		procedure	UMLS:SNOMED:77166000	Percutaneous balloon valvuloplasty of aortic valve
cannot have		procedure	UMLS:CPT:33410	Replacement, aortic valve, open, with cardiopulmonary bypass; with stentless tissue valve
	or	procedure	UMLS:CPT:33405	Replacement, aortic valve, open, with cardiopulmonary bypass; with prosthetic valve other than homograft or stentless valve
	or	procedure	UMLS:CPT:1029693	Replacement, aortic valve, open, with cardiopulmonary bypass
	or	procedure	UMLS:CPT:33405	Replacement, aortic valve, open, with cardiopulmonary bypass; with prosthetic valve other than homograft or stentless valve
	or	procedure	UMLS:CPT:33406	Replacement, aortic valve, open, with cardiopulmonary bypass; with allograft valve (freehand)
	or	procedure	UMLS:CPT:33410	Replacement, aortic valve, open, with cardiopulmonary bypass; with stentless tissue valve
	or	procedure	UMLS:ICD10PCS:02RF08Z	Replacement of Aortic Valve with Zooplastic Tissue, Open Approach
	or	procedure	UMLS:ICD10PCS:02RF0JZ	Replacement of Aortic Valve with Synthetic Substitute, Open Approach
	or	procedure	UMLS:ICD10PCS:02RF0KZ	Replacement of Aortic Valve with Nonautologous Tissue Substitute, Open Approach
	or	procedure	UMLS:ICD10PCS:02RF07Z	Replacement of Aortic Valve with Autologous Tissue Substitute, Open Approach
	or	procedure	UMLS:ICD10PCS:02RF08N	Replacement of Aortic Valve with Zooplastic Tissue, using Rapid Deployment Technique, Open Approach

	or	procedure	UMLS:ICD10PCS:X2RF032	Replacement of Aortic Valve using Zooplastic Tissue, Rapid Deployment Technique, Open Approach, New Technology Group 2 (deprecated 2022)
date constraint		The terms in this group occurred at any time		
event relationship		Any instance of Group 1B occurred within 1 day and 1 month after any instance of Group 1A		
**Group 1B**				
must have	any of	procedure	UMLS:CPT:33363	Transcatheter aortic valve replacement (TAVR/TAVI) with prosthetic valve; open axillary artery approach
		procedure	UMLS:CPT:33362	Transcatheter aortic valve replacement (TAVR/TAVI) with prosthetic valve; open femoral artery approach
		procedure	UMLS:CPT:33366	Transcatheter aortic valve replacement (TAVR/TAVI) with prosthetic valve; transapical exposure (eg, left thoracotomy)
		procedure	UMLS:CPT:33361	Transcatheter aortic valve replacement (TAVR/TAVI) with prosthetic valve; percutaneous femoral artery approach
		procedure	UMLS:CPT:33364	Transcatheter aortic valve replacement (TAVR/TAVI) with prosthetic valve; open iliac artery approach
		procedure	UMLS:CPT:33365	Transcatheter aortic valve replacement (TAVR/TAVI) with prosthetic valve; transaortic approach (eg, median sternotomy, mediastinotomy)
		procedure	UMLS:SNOMED:773996000	Transcatheter aortic valve implantation
		procedure	UMLS:SNOMED:725351001	Transcatheter aortic valve replacement (deprecated 2022)
		procedure	UMLS:CPT:1021150	Transcatheter aortic valve replacement (TAVR/TAVI) with prosthetic valve
		procedure	UMLS:CPT:33368	Transcatheter aortic valve replacement (TAVR/TAVI) with prosthetic valve; cardiopulmonary bypass support with open peripheral arterial and venous cannulation (eg, femoral, iliac, axillary vessels) (List separately in addition to code for primary procedure)
		procedure	UMLS:CPT:33369	Transcatheter aortic valve replacement (TAVR/TAVI) with prosthetic valve; cardiopulmonary bypass support with central arterial and venous cannulation (eg, aorta, right atrium, pulmonary artery) (List separately in addition to code for primary procedure)
		procedure	UMLS:CPT:33367	Transcatheter aortic valve replacement (TAVR/TAVI) with prosthetic valve; cardiopulmonary bypass support with percutaneous peripheral arterial and venous cannulation (eg, femoral vessels) (List separately in addition to code for primary procedure)

Analysis Setup

This section contains the Index Event and Time Window definitions and a list of selected outcomes and the analyses.

Index Event & Time Window Definitions

The index event defines the point in time when each patient in the cohort enters the analysis. To define an index event for the cohort, one or more criteria for the cohort must be selected. The index date for each patient within a cohort is the day on which the patient first met the selected criteria for the cohort (listed in the table below).

As the index event defines the earliest time point after which outcomes are analyzed, the time window defines the duration during which outcomes are analyzed. The time window can start on the same day as the index event or at any specified time interval after the index event. The time window can end any time after the start date. Outcomes are defined as diagnoses, medications, procedures, or laboratory values that happened in the time window starting after the first occurrence of the index event.

Time Window Used in this Analysis

This analysis included outcomes that occurred in the time window that started 1 day after the first occurrence of the index event and ended 30 days after the first occurrence of the index event.

The index event only includes events that occurred up to 20 years ago. Patients whose index event occurred 20 years or more ago are excluded. In this analysis, 0 patients in Cohort 1 and 0 patients in Cohort 2 were excluded because they met the index event more than 20 years ago.

Index Events Used in this Analysis

Index events for the Compare Outcomes analysis were derived from the cohort definitions. Index events were defined separately for each cohort and were based on the criteria used in the original cohort definition. Please see Appendix B for the text representation of the index event definition.

The index event for Cohort 1 (query name: TAVR Direct Final) was defined as the following:

**Group 1**					
	**Group 1A**				
	must have		diagnosis	UMLS:ICD10CM:I35.0	Nonrheumatic aortic (valve) stenosis
		and	diagnosis	UMLS:ICD10CM:R57.0	Cardiogenic shock
	cannot have		procedure	UMLS:CPT:33410	Replacement, aortic valve, open, with cardiopulmonary bypass; with stentless tissue valve
		or	procedure	UMLS:CPT:33405	Replacement, aortic valve, open, with cardiopulmonary bypass; with prosthetic valve other than homograft or stentless valve
		or	procedure	UMLS:ICD10PCS:02RF0JZ	Replacement of Aortic Valve with Synthetic Substitute, Open Approach
		or	procedure	UMLS:CPT:33405	Replacement, aortic valve, open, with cardiopulmonary bypass; with prosthetic valve other than homograft or stentless valve
		or	procedure	UMLS:CPT:33411	Replacement, aortic valve; with aortic annulus enlargement, noncoronary sinus

	or	procedure	UMLS:ICD10PCS:02RF08Z	Replacement of Aortic Valve with Zooplastic Tissue, Open Approach
	or	procedure	UMLS:ICD10PCS:02RF0KZ	Replacement of Aortic Valve with Nonautologous Tissue Substitute, Open Approach
	or	procedure	UMLS:ICD10PCS:02RF07Z	Replacement of Aortic Valve with Autologous Tissue Substitute, Open Approach
	or	procedure	UMLS:CPT:33413	Replacement, aortic valve; by translocation of autologous pulmonary valve with allograft replacement of pulmonary valve (Ross procedure)
date constraint		The terms in this group occurred at any time		
event relationship		Any instance of Group 1B occurred within 10 days on or after any instance of Group 1A		
**Group 1B**				
must have	any of	procedure	UMLS:CPT:33363	Transcatheter aortic valve replacement (TAVR/TAVI) with prosthetic valve; open axillary artery approach
		procedure	UMLS:CPT:33362	Transcatheter aortic valve replacement (TAVR/TAVI) with prosthetic valve; open femoral artery approach
		procedure	UMLS:CPT:33361	Transcatheter aortic valve replacement (TAVR/TAVI) with prosthetic valve; percutaneous femoral artery approach
		procedure	UMLS:CPT:33364	Transcatheter aortic valve replacement (TAVR/TAVI) with prosthetic valve; open iliac artery approach
		procedure	UMLS:SNOMED:773996000	Transcatheter aortic valve implantation
		procedure	UMLS:CPT:1021150	Transcatheter aortic valve replacement (TAVR/TAVI) with prosthetic valve
		procedure	UMLS:CPT:33368	Transcatheter aortic valve replacement (TAVR/TAVI) with prosthetic valve; cardiopulmonary bypass support with open peripheral arterial and venous cannulation (eg, femoral, iliac, axillary vessels) (List separately in addition to code for primary procedure)
		procedure	UMLS:CPT:33369	Transcatheter aortic valve replacement (TAVR/TAVI) with prosthetic valve; cardiopulmonary bypass support with central arterial and venous cannulation (eg, aorta, right atrium, pulmonary artery) (List separately in addition to code for primary procedure)
		procedure	UMLS:CPT:33367	Transcatheter aortic valve replacement (TAVR/TAVI) with prosthetic valve; cardiopulmonary bypass support with percutaneous peripheral arterial and venous cannulation (eg, femoral vessels) (List separately in addition to code for primary procedure)

The index event for Cohort 2 (query name: BAV TAVR Final) was defined as the following:

**Group 1**				
**Group 1A**				
must have		diagnosis	UMLS:ICD10CM:I35.0	Nonrheumatic aortic (valve) stenosis

	and	diagnosis	UMLS:ICD10CM:R57.0	Cardiogenic shock
	and any of	procedure	UMLS:CPT:92986	Percutaneous balloon valvuloplasty; aortic valve
		procedure	UMLS:ICD10PCS:027F3ZZ	Dilation of Aortic Valve, Percutaneous Approach
		procedure	UMLS:CPT:1012998	Percutaneous balloon valvuloplasty
		procedure	UMLS:ICD10PCS:027F4ZZ	Dilation of Aortic Valve, Percutaneous Endoscopic Approach
		procedure	UMLS:CPT:92986	Percutaneous balloon valvuloplasty; aortic valve
		procedure	UMLS:SNOMED:77166000	Percutaneous balloon valvuloplasty of aortic valve
cannot have		procedure	UMLS:CPT:33410	Replacement, aortic valve, open, with cardiopulmonary bypass; with stentless tissue valve
	or	procedure	UMLS:CPT:33405	Replacement, aortic valve, open, with cardiopulmonary bypass; with prosthetic valve other than homograft or stentless valve
	or	procedure	UMLS:CPT:1029693	Replacement, aortic valve, open, with cardiopulmonary bypass
	or	procedure	UMLS:CPT:33405	Replacement, aortic valve, open, with cardiopulmonary bypass; with prosthetic valve other than homograft or stentless valve
	or	procedure	UMLS:CPT:33406	Replacement, aortic valve, open, with cardiopulmonary bypass; with allograft valve (freehand)
	or	procedure	UMLS:CPT:33410	Replacement, aortic valve, open, with cardiopulmonary bypass; with stentless tissue valve
	or	procedure	UMLS:ICD10PCS:02RF08Z	Replacement of Aortic Valve with Zooplastic Tissue, Open Approach
	or	procedure	UMLS:ICD10PCS:02RF0JZ	Replacement of Aortic Valve with Synthetic Substitute, Open Approach
	or	procedure	UMLS:ICD10PCS:02RF0KZ	Replacement of Aortic Valve with Nonautologous Tissue Substitute, Open Approach
	or	procedure	UMLS:ICD10PCS:02RF07Z	Replacement of Aortic Valve with Autologous Tissue Substitute, Open Approach
	or	procedure	UMLS:ICD10PCS:02RF08N	Replacement of Aortic Valve with Zooplastic Tissue, using Rapid Deployment Technique, Open Approach
	or	procedure	UMLS:ICD10PCS:X2RF032	Replacement of Aortic Valve using Zooplastic Tissue, Rapid Deployment Technique, Open Approach, New Technology Group 2 (deprecated 2022)
date constraint		The terms in this group occurred at any time		
event relationship		Any instance of Group 1B occurred within 1 day and 1 month after any instance of Group 1A		
**Group 1B**				
must have	any of	procedure	UMLS:CPT:33363	Transcatheter aortic valve replacement (TAVR/TAVI) with prosthetic valve; open axillary artery approach
		procedure	UMLS:CPT:33362	Transcatheter aortic valve replacement (TAVR/TAVI) with prosthetic valve; open femoral artery approach

procedure	UMLS:CPT:33366	Transcatheter aortic valve replacement (TAVR/TAVI) with prosthetic valve; transapical exposure (eg, left thoracotomy)
procedure	UMLS:CPT:33361	Transcatheter aortic valve replacement (TAVR/TAVI) with prosthetic valve; percutaneous femoral artery approach
procedure	UMLS:CPT:33364	Transcatheter aortic valve replacement (TAVR/TAVI) with prosthetic valve; open iliac artery approach
procedure	UMLS:CPT:33365	Transcatheter aortic valve replacement (TAVR/TAVI) with prosthetic valve; transaortic approach (eg, median sternotomy, mediastinotomy)
procedure	UMLS:SNOMED:773996000	Transcatheter aortic valve implantation
procedure	UMLS:SNOMED:725351001	Transcatheter aortic valve replacement (deprecated 2022)
procedure	UMLS:CPT:1021150	Transcatheter aortic valve replacement (TAVR/TAVI) with prosthetic valve
procedure	UMLS:CPT:33368	Transcatheter aortic valve replacement (TAVR/TAVI) with prosthetic valve; cardiopulmonary bypass support with open peripheral arterial and venous cannulation (eg, femoral, iliac, axillary vessels) (List separately in addition to code for primary procedure)
procedure	UMLS:CPT:33369	Transcatheter aortic valve replacement (TAVR/TAVI) with prosthetic valve; cardiopulmonary bypass support with central arterial and venous cannulation (eg, aorta, right atrium, pulmonary artery) (List separately in addition to code for primary procedure)
procedure	UMLS:CPT:33367	Transcatheter aortic valve replacement (TAVR/TAVI) with prosthetic valve; cardiopulmonary bypass support with percutaneous peripheral arterial and venous cannulation (eg, femoral vessels) (List separately in addition to code for primary procedure)

Analyses Specifications

The Compare Outcomes Analytic supports four types of analyses: Measure of Association, Survival, Number of Instances, and Lab result distribution. The first three analyses support the “exclude patients with outcomes prior to the window” setting. This option can exclude patients from the analysis if they are not at risk for an outcome (e.g., if the outcome is a chronic disease). When "exclude patients with the outcome prior to the time window" is not checked, all patients in the cohort are included in the analysis, regardless of whether they had the outcome prior to the time window. When "exclude patients with the outcome prior to the time window" is checked, patients are excluded from the analysis if their record includes the outcome prior to the beginning of the time window. This selection will exclude all patients with the outcome prior to the index event. If the start of the time window for the analysis falls some days after the index event, patients will also be excluded if they have the outcome between the index event and the start of the time window.

Measure of Association Analysis

The Measure of Association Analysis calculates and compares the fraction of patients with the selected outcome. The output summary includes: Patients in each Cohort (count of patients meeting query criteria); Patients with Outcome in each Cohort (of the patients in the cohort, count of patients that had the outcome in the time window); and Risk (the fraction of patients in the cohort that have the outcome in the time window, i.e. Patients with Outcome / Patients in Cohort). In addition, Risk Difference (the difference in the risks in Cohort 1 and Cohort 2), Risk Ratio (the ratio of the risks in Cohort 1 and Cohort 2), and Odds Ratio (the ratio of the odds in Cohort 1 and Cohort 2). The bar chart shows the risk of the outcome for the both cohorts.

Survival Analysis

The Kaplan-Meier Analysis estimates probability of the outcome at a respective time interval (daily time interval is used in this analysis). In order to account for the patients who exited the cohort during the analysis period, and therefore should not be included in the analysis, censoring is applied. In this analysis, patients are removed from the analysis (censored) after the last fact in their record.

The output summary includes: Patients in each Cohort (count of patients meeting query criteria); Patients with Outcome (of the patients in the cohort, count of patients that had the outcome in the time window); Median Survival (the number of days when the survival drops below 50%; the "-" indicates that survival does not drop below 50% during the time window); and Survival Probability at End of Time Window (the % survival at the end of the time window). In addition, Log-Rank test, Hazard Ratio and test for Proportionality.

Number of Instances Analysis

The Number of Instances Analysis calculates how many times the outcome occurred in the time window. This analysis includes two additional settings: include patients with zero instances; the definition of an instance.

Selecting to exclude patients with zero instances will remove these patients from the calculations for mean number of instances, standard deviation, or median. The histogram showing the distribution of patients by number of instances will not contain a bar for zero. Alternatively, by selecting to include patients with zero instances, the mean, standard deviation, and median for number of instances will reflect these patients. The histogram will contain a bar for zero patients.

The definition of an instance affects how counts are analyzed. By selecting Date, each calendar date on which any of the terms selected in the outcome are recorded will represent one instance. For example, if the outcome is "Med A or Med B," and a patient has "Med A" on January 3, then both medications on January 4, then "Med B" on January 6, then that patient is considered to have three instances– January 3, January 4, and January 6. Note that if an outcome occurs across several dates (e.g. Visit: inpatient encounter), then only the start date is tracked for the purpose of counting instances. A patient who begins at stay on January 1, ends that stay on January 3, begins another stay on January 10, and ends that stay on January 15, is considered to have two instances of the outcome.

Selecting Visit as an Instance will count any visit that includes the outcome as one instance, regardless of how many times it occurred. For instance, consider a patient administered an analgesic on each of the three days that make up an inpatient stay following some index event. If analgesic is an outcome, these three administrations will represent only one instance, because all three are associated with the same visit.

The output summary includes: Patients in Cohort (count of patients meeting query criteria); Patients with Outcome (of the patients in the cohort, count of patients that had the outcome in the time window); Mean (mean of the counts); Standard Deviation (standard deviation of the counts); Median (median of the counts);

and Median (1+ instances) when patients with zero instances included in the analysis. In addition, T-Test statistics testing for the difference between the cohorts is included.

Laboratory Results Analysis

Lab Results can be included in the analysis only for the outcomes that are labs. Only the most recent lab values in the time window are included. For the lab results that are numeric, the outcome summary includes: Patients in Cohort (count of patients meeting query criteria); Patients with Outcome (of the patients in the cohort, count of patients that had the outcome in the time window); Mean (mean of the counts); and Standard Deviation (the standard deviation for lab values across patients in the cohort). In addition, T-Test statistics testing for the difference between the cohorts is included.

For the non-numeric lab results, three values are reported: counts of Negative; Positives; and Unknowns. The counts are represented in the bar chart as percentages of the total counts.

Outcome Definitions

Table below outlines the definitions for each outcome and the analysis specifications. For outcome definitions consisting of more than one term, at least one term must match. Please see Appendix C for the text representation of the outcome definitions.

**Mortality**		
**Outcome definition**		
Demographics	Deceased	Deceased
Diagnosis	UMLS:ICD10CM:R99	Ill-defined and unknown cause of mortality
Diagnosis	UMLS:ICD10CM:R99-R99	Ill-defined and unknown cause of mortality (R99)
Diagnosis	UMLS:ICD10CM:R69	Illness, unspecified
**Settings for the performed analyses**		
Risk analysis		excluding patients with outcome prior to the time window
Kaplan - Meier survival analysis		excluding patients with outcome prior to the time window
**Pacemaker**		
**Outcome definition**		
Procedure	UMLS:CPT:33208	Insertion of new or replacement of permanent pacemaker with transvenous electrode(s); atrial and ventricular
Procedure	UMLS:CPT:33207	Insertion of new or replacement of permanent pacemaker with transvenous electrode(s); ventricular
Procedure	UMLS:CPT:33274	Transcatheter insertion or replacement of permanent leadless pacemaker, right ventricular, including imaging guidance (eg, fluoroscopy, venous ultrasound, ventriculography, femoral venography) and device evaluation (eg, interrogation or programming), when performed
Procedure	UMLS:CPT:1037892	Dual-Chamber Leadless Pacemaker
**Settings for the performed analyses**		
Kaplan - Meier survival analysis		excluding patients with outcome prior to the time window
Risk analysis		excluding patients with outcome prior to the time window
**MACE**		
**Outcome definition**		
Demographics	Deceased	Deceased
Diagnosis	UMLS:ICD10CM:I21.0	ST elevation (STEMI) myocardial infarction of anterior wall
Diagnosis	UMLS:ICD10CM:I21.1	ST elevation (STEMI) myocardial infarction of inferior wall
Diagnosis	UMLS:ICD10CM:I21.2	ST elevation (STEMI) myocardial infarction of other sites
Diagnosis	UMLS:ICD10CM:I21.3	ST elevation (STEMI) myocardial infarction of unspecified site
Diagnosis	UMLS:ICD10CM:I21.4	Non-ST elevation (NSTEMI) myocardial infarction
Diagnosis	UMLS:ICD10CM:I21.9	Acute myocardial infarction, unspecified
Diagnosis	UMLS:ICD9CM:410	Acute myocardial infarction
Diagnosis	UMLS:ICD10CM:I63.0	Cerebral infarction due to thrombosis of precerebral arteries

	Diagnosis	UMLS:ICD10CM:I63.1	Cerebral infarction due to embolism of precerebral arteries
	Diagnosis	UMLS:ICD10CM:I63.2	Cerebral infarction due to unspecified occlusion or stenosis of precerebral arteries
	Diagnosis	UMLS:ICD10CM:I63.3	Cerebral infarction due to thrombosis of cerebral arteries
	Diagnosis	UMLS:ICD10CM:I63.4	Cerebral infarction due to embolism of cerebral arteries
	Diagnosis	UMLS:ICD10CM:I63.5	Cerebral infarction due to unspecified occlusion or stenosis of cerebral arteries
	Diagnosis	UMLS:ICD10CM:I63.8	Other cerebral infarction
**Settings for the performed analyses**			
	Risk analysis		including patients with outcome prior to the time window
	Kaplan - Meier survival analysis		including patients with outcome prior to the time window
**Hemodialysis**			
**Outcome definition**			
	Procedure	UMLS:ICD9CM:39.95	Hemodialysis
	Procedure	UMLS:SNOMED:302497006	Hemodialysis
	Procedure	UMLS:CPT:1012752	Hemodialysis Procedures
**Settings for the performed analyses**			
	Risk analysis		including patients with outcome prior to the time window
	Kaplan - Meier survival analysis		including patients with outcome prior to the time window
**Atrial fibrillation**			
**Outcome definition**			
	Diagnosis	UMLS:ICD10CM:I48.0	Paroxysmal atrial fibrillation
	Diagnosis	UMLS:ICD10CM:I48.1	Persistent atrial fibrillation
**Settings for the performed analyses**			
	Risk analysis		including patients with outcome prior to the time window
	Kaplan - Meier survival analysis		including patients with outcome prior to the time window
**HF**			
**Outcome definition**			
	Diagnosis	UMLS:ICD10CM:I50	Heart failure
	Diagnosis	UMLS:ICD10CM:I50.1	Left ventricular failure, unspecified
	Diagnosis	UMLS:ICD10CM:I50.2	Systolic (congestive) heart failure
	Diagnosis	UMLS:ICD10CM:I50.3	Diastolic (congestive) heart failure
	Diagnosis	UMLS:ICD10CM:I50.4	Combined systolic (congestive) and diastolic (congestive) heart failure
	Diagnosis	UMLS:ICD10CM:I50.8	Other heart failure
	Diagnosis	UMLS:ICD10CM:I50.9	Heart failure, unspecified
	Diagnosis	UMLS:ICD9CM:428	Heart failure
**Settings for the performed analyses**			
	Kaplan - Meier survival analysis		including patients with outcome prior to the time window
	Risk analysis		including patients with outcome prior to the time window
**AMI**			
**Outcome definition**			
	Diagnosis	UMLS:ICD10CM:I21	Acute myocardial infarction
	Diagnosis	UMLS:ICD10CM:I21.0	ST elevation (STEMI) myocardial infarction of anterior wall
	Diagnosis	UMLS:ICD10CM:I21.1	ST elevation (STEMI) myocardial infarction of inferior wall
	Diagnosis	UMLS:ICD10CM:I21.2	ST elevation (STEMI) myocardial infarction of other sites
	Diagnosis	UMLS:ICD10CM:I21.3	ST elevation (STEMI) myocardial infarction of unspecified site
	Diagnosis	UMLS:ICD10CM:I21.4	Non-ST elevation (NSTEMI) myocardial infarction
	Diagnosis	UMLS:ICD10CM:I21.9	Acute myocardial infarction, unspecified
	Diagnosis	UMLS:ICD9CM:410	Acute myocardial infarction
**Settings for the performed analyses**			
	Risk analysis		including patients with outcome prior to the time window
	Kaplan - Meier survival analysis		including patients with outcome prior to the time window
**Cerebral Infraction**			
**Outcome definition**			
	Diagnosis	UMLS:ICD10CM:I63	Cerebral infarction
	Diagnosis	UMLS:ICD10CM:I63.0	Cerebral infarction due to thrombosis of precerebral arteries

Diagnosis	UMLS:ICD10CM:I63.1	Cerebral infarction due to embolism of precerebral arteries
Diagnosis	UMLS:ICD10CM:I63.2	Cerebral infarction due to unspecified occlusion or stenosis of precerebral arteries
Diagnosis	UMLS:ICD10CM:I63.3	Cerebral infarction due to thrombosis of cerebral arteries
Diagnosis	UMLS:ICD10CM:I63.4	Cerebral infarction due to embolism of cerebral arteries
Diagnosis	UMLS:ICD10CM:I63.5	Cerebral infarction due to unspecified occlusion or stenosis of cerebral arteries
Diagnosis	UMLS:ICD10CM:I63.9	Cerebral infarction, unspecified
**Settings for the performed analyses**		
Risk analysis		including patients with outcome prior to the time window
Kaplan - Meier survival analysis		including patients with outcome prior to the time window

Propensity Score Matching

Propensity score matching was performed on 28 characteristic(s). In the Demographics category patients were matched on Age at Index, Male, Female, White, Black or African American, Asian characteristic(s). In the Diagnosis category patients were matched on Hypertensive diseases, Ischemic heart diseases, Cerebrovascular diseases, Heart failure, Atrial fibrillation and flutter, Cardiac arrest, Diabetes mellitus, Malnutrition, Overweight, obesity and other hyperalimentation, Chronic kidney disease (CKD) characteristic(s). In the Procedure category patients were matched on Intubation, endotracheal, emergency procedure, Cardiac Assist Procedures, Extracorporeal Membrane Oxygenation or Extracorporeal Life Support Services and Procedures characteristic(s). In the Medication category patients were matched on SYMPATHOMIMETICS (ADRENERGICS) characteristic(s). In the Laboratory category patients were matched on Creatinine [Mass/volume] in Serum, Plasma or Blood, Hemoglobin [Mass/volume] in Blood, Albumin [Mass/volume] in Serum, Plasma or Blood, Natriuretic peptide B [Mass/volume] in Serum, Plasma or Blood, Troponin I.cardiac [Mass/volume] in Serum, Plasma or Blood, Hemoglobin A1c/Hemoglobin.total in Blood, BMI, Left Ventricular Ejection Fraction (LVEF) (%) characteristic(s). Characteristics of the cohorts before and after matching are summarized in the table below.

**Cohort 1 and cohort 2 patient count before and after propensity score matching**		
Cohort	Patient count before matching	Patient count after matching
1 - TAVR Direct Final	1,701	198
2 - BAV TAVR Final	201	198

Propensity score density function - Before and after matching (cohort 1 - purple, cohort 2 - green)

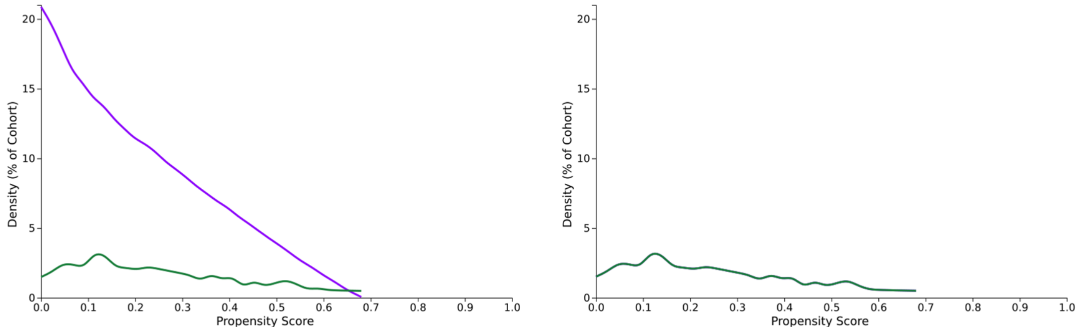

Cohort 1 (N = 1,701) and cohort 2 (N = 201) characteristics before propensity score matching

Demographics

Cohort			Mean ± SD	Patients	% of Cohort	P-Value	Std diff.
1<br>2	AI	Age at Index	75.5 +/- 10.6<br>76.4 +/- 9.3	1,701<br>201	100%<br>100%	0.231	0.094

1<br>2	2106-3	White		1,347<br>145	79.2%<br>72.1%	0.022	0.165
1<br>2	1002-5	American Indian or Alaska Native		10<br>10	0.6%<br>5.0%	<0.001	0.269
1<br>2	UNK	Unknown Race		168<br>32	9.9%<br>15.9%	0.008	0.181
1<br>2	F	Female		604<br>63	35.5%<br>31.3%	0.242	0.088
1<br>2	2076-8	Native Hawaiian or Other Pacific Islander		10<br>10	0.6%<br>5.0%	<0.001	0.269
1<br>2	2054-5	Black or African American		99<br>10	5.8%<br>5.0%	0.626	0.037
1<br>2	M	Male		1,003<br>112	59.0%<br>55.7%	0.377	0.066
1<br>2	2131-1	Other Race		27<br>10	1.6%<br>5.0%	0.001	0.191
1<br>2	2028-9	Asian		46<br>11	2.7%<br>5.5%	0.029	0.140

Diagnosis

Cohort			Mean ± SD	Patients	% of Cohort	P-Value	Std diff.
1<br>2	I10-I1A	Hypertensive diseases		1,344<br>171	79.0%<br>85.1%	0.043	0.158
1<br>2	I20-I25	Ischemic heart diseases		1,323<br>175	77.8%<br>87.1%	0.002	0.246
1<br>2	I60-I69	Cerebrovascular diseases		376<br>43	22.1%<br>21.4%	0.818	0.017
1<br>2	I50	Heart failure		1,372<br>196	80.7%<br>97.5%	<0.001	0.561
1<br>2	I48	Atrial fibrillation and flutter		858<br>123	50.4%<br>61.2%	0.004	0.218
1<br>2	I46	Cardiac arrest		157<br>26	9.2%<br>12.9%	0.092	0.118
1<br>2	E08-E13	Diabetes mellitus		698<br>94	41.0%<br>46.8%	0.119	0.116
1<br>2	E40-E46	Malnutrition		255<br>54	15.0%<br>26.9%	<0.001	0.295
1<br>2	E65-E68	Overweight, obesity and other hyperalimentation		393<br>44	23.1%<br>21.9%	0.699	0.029
1<br>2	N18	Chronic kidney disease (CKD)		803<br>108	47.2%<br>53.7%	0.080	0.131

Procedure

Cohort			Mean ± SD	Patients	% of Cohort	P-Value	Std diff.
1<br>2	31500	Intubation, endotracheal, emergency procedure		92<br>29	5.4%<br>14.4%	<0.001	0.305
1<br>2	1006339	Cardiac Assist Procedures		147<br>64	8.6%<br>31.8%	<0.001	0.603
1<br>2	1021846	Extracorporeal Membrane Oxygenation or Extracorporeal Life Support Services and Procedures		26<br>10	1.5%<br>5.0%	0.001	0.195

Medication

Cohort			Mean ± SD	Patients	% of Cohort	P-Value	Std diff.
1<br>2	AU100	SYMPATHOMIMETICS (ADRENERGICS)		756<br>163	44.4%<br>81.1%	<0.001	0.819

Laboratory

Cohort			Mean ± SD	Patients	% of Cohort	P-Value	Std diff.
1<br>2	9024	Creatinine [Mass/volume] in Serum, Plasma or Blood	1.6 +/- 1.4<br>1.6 +/- 1.5	1,531<br>197	90.0%<br>98.0%	0.913	0.008
1<br>2		0 - 0 mg/dL		1,531<br>197	90.0%<br>98.0%	<0.001	0.342
1<br>2	9014	Hemoglobin [Mass/volume] in Blood	11.1 +/- 2.3<br>9.7 +/- 2.4	1,528<br>197	89.8%<br>98.0%	<0.001	0.581
1<br>2		0 - 0 g/dL		1,528<br>197	89.8%<br>98.0%	<0.001	0.347
1<br>2	9045	Albumin [Mass/volume] in Serum, Plasma or Blood	3.4 +/- 0.6<br>3.1 +/- 0.5	1,412<br>186	83.0%<br>92.5%	<0.001	0.577
1<br>2		0 - 0 g/dL		1,412<br>186	83.0%<br>92.5%	<0.001	0.294
1<br>2	9003	Natriuretic peptide B [Mass/volume] in Serum, Plasma or Blood	3787.7 +/- 8156.3<br>8133.7 +/- 12970.1	650<br>77	38.2%<br>38.3%	<0.001	0.401
1<br>2		0 - 0 pg/mL		650<br>77	38.2%<br>38.3%	0.979	0.002
1<br>2	9005	Troponin I.cardiac [Mass/volume] in Serum, Plasma or Blood	4.1 +/- 25.1<br>5.2 +/- 8.1	254<br>41	14.9%<br>20.4%	0.776	0.060
1<br>2		0 - 0 ng/mL		254<br>41	14.9%<br>20.4%	0.043	0.144
1<br>2	9037	Hemoglobin A1c/Hemoglobin.total in Blood	6.4 +/- 1.4<br>6.2 +/- 1.2	679<br>112	39.9%<br>55.7%	0.313	0.108
1<br>2		0 - 0 %		679<br>112	39.9%<br>55.7%	<0.001	0.320
1<br>2	9083	BMI	28.5 +/- 6.7<br>28.1 +/- 6.3	1,192<br>142	70.1%<br>70.6%	0.481	0.064
1<br>2		0 - 0 kg/m2		1,193<br>142	70.1%<br>70.6%	0.881	0.011
1<br>2	2003	Left Ventricular Ejection Fraction (LVEF) (%)	37.2 +/- 17.6<br>35.0 +/- 17.5	242<br>66	14.2%<br>32.8%	0.353	0.130
1<br>2		0 - 0 %		242<br>66	14.2%<br>32.8%	<0.001	0.450

Cohort 1 (N = 198) and cohort 2 (N = 198) characteristics after propensity score matching

Demographics

Cohort			Mean ± SD	Patients	% of Cohort	P-Value	Std diff.
1<br>2	AI	Age at Index	76.4 +/- 9.8<br>76.3 +/- 9.2	198<br>198	100%<br>100%	0.962	0.005
1<br>2	2106-3	White		144<br>144	72.7%<br>72.7%	1	<0.001

Cohort			Mean ± SD	Patients	% of Cohort	P-Value	Std diff.
1<br>2	1002-5	American Indian or Alaska Native		10<br>10	5.1%<br>5.1%	1	<0.001
1<br>2	UNK	Unknown Race		32<br>30	16.2%<br>15.2%	0.782	0.028
1<br>2	F	Female		59<br>62	29.8%<br>31.3%	0.743	0.033
1<br>2	2076-8	Native Hawaiian or Other Pacific Islander		10<br>10	5.1%<br>5.1%	1	<0.001
1<br>2	2054-5	Black or African American		11<br>10	5.6%<br>5.1%	0.823	0.023
1<br>2	M	Male		114<br>112	57.6%<br>56.6%	0.839	0.020
1<br>2	2131-1	Other Race		10<br>10	5.1%<br>5.1%	1	<0.001
1<br>2	2028-9	Asian		10<br>11	5.1%<br>5.6%	0.823	0.023

Diagnosis

Cohort			Mean ± SD	Patients	% of Cohort	P-Value	Std diff.
1<br>2	I10-I1A	Hypertensive diseases		167<br>169	84.3%<br>85.4%	0.779	0.028
1<br>2	I20-I25	Ischemic heart diseases		172<br>172	86.9%<br>86.9%	1	<0.001
1<br>2	I60-I69	Cerebrovascular diseases		51<br>43	25.8%<br>21.7%	0.345	0.095
1<br>2	I50	Heart failure		193<br>193	97.5%<br>97.5%	1	<0.001
1<br>2	I48	Atrial fibrillation and flutter		120<br>121	60.6%<br>61.1%	0.918	0.010
1<br>2	I46	Cardiac arrest		37<br>26	18.7%<br>13.1%	0.131	0.152
1<br>2	E08-E13	Diabetes mellitus		97<br>92	49.0%<br>46.5%	0.615	0.051
1<br>2	E40-E46	Malnutrition		53<br>53	26.8%<br>26.8%	1	<0.001
1<br>2	E65-E68	Overweight, obesity and other hyperalimentation		47<br>44	23.7%<br>22.2%	0.720	0.036
1<br>2	N18	Chronic kidney disease (CKD)		106<br>107	53.5%<br>54.0%	0.920	0.010

Procedure

Cohort			Mean ± SD	Patients	% of Cohort	P-Value	Std diff.
1<br>2	31500	Intubation, endotracheal, emergency procedure		26<br>28	13.1%<br>14.1%	0.770	0.029
1<br>2	1006339	Cardiac Assist Procedures		63<br>62	31.8%<br>31.3%	0.914	0.011
1<br>2	1021846	Extracorporeal Membrane Oxygenation or Extracorporeal Life Support Services and Procedures		10<br>10	5.1%<br>5.1%	1	<0.001

Medication

Cohort			Mean ± SD	Patients	% of Cohort	P-Value	Std diff.

Cohort			Mean ± SD	Patients	% of Cohort	P-Value	Std diff.
1<br>2	AU100	SYMPATHOMIMETICS (ADRENERGICS)		161<br>160	81.3%<br>80.8%	0.898	0.013

Laboratory

Cohort			Mean ± SD	Patients	% of Cohort	P-Value	Std diff.
1<br>2	9024	Creatinine [Mass/volume] in Serum, Plasma or Blood	1.7 +/- 1.4<br>1.6 +/- 1.5	192<br>194	97.0%<br>98.0%	0.953	0.006
1<br>2		0 - 0 mg/dL		192<br>194	97.0%<br>98.0%	0.522	0.064
1<br>2	9014	Hemoglobin [Mass/volume] in Blood	10.0 +/- 2.5<br>9.8 +/- 2.4	193<br>194	97.5%<br>98.0%	0.332	0.099
1<br>2		0 - 0 g/dL		193<br>194	97.5%<br>98.0%	0.736	0.034
1<br>2	9045	Albumin [Mass/volume] in Serum, Plasma or Blood	3.1 +/- 0.5<br>3.1 +/- 0.5	184<br>183	92.9%<br>92.4%	0.684	0.042
1<br>2		0 - 0 g/dL		184<br>183	92.9%<br>92.4%	0.847	0.019
1<br>2	9003	Natriuretic peptide B [Mass/volume] in Serum, Plasma or Blood	7839.0 +/- 11747.1<br>7856.1 +/- 13028.6	73<br>75	36.9%<br>37.9%	0.993	0.001
1<br>2		0 - 0 pg/mL		73<br>75	36.9%<br>37.9%	0.835	0.021
1<br>2	9005	Troponin I.cardiac [Mass/volume] in Serum, Plasma or Blood	5.4 +/- 9.7<br>5.2 +/- 8.1	47<br>41	23.7%<br>20.7%	0.915	0.023
1<br>2		0 - 0 ng/mL		47<br>41	23.7%<br>20.7%	0.468	0.073
1<br>2	9037	Hemoglobin A1c/Hemoglobin.total in Blood	6.3 +/- 1.6<br>6.2 +/- 1.2	110<br>110	55.6%<br>55.6%	0.446	0.103
1<br>2		0 - 0 %		110<br>110	55.6%<br>55.6%	1	<0.001
1<br>2	9083	BMI	28.3 +/- 6.5<br>28.2 +/- 6.3	140<br>141	70.7%<br>71.2%	0.881	0.018
1<br>2		0 - 0 kg/m2		140<br>141	70.7%<br>71.2%	0.912	0.011
1<br>2	2003	Left Ventricular Ejection Fraction (LVEF) (%)	32.7 +/- 14.8<br>35.2 +/- 17.7	65<br>64	32.8%<br>32.3%	0.393	0.151
1<br>2		0 - 0 %		65<br>64	32.8%<br>32.3%	0.915	0.011

Results

Results are summarized in the tables below. Outcomes analysis was performed on the cohorts after propensity score matching.

Follow-up Time (Before Matching)

Cohort	Mean Follow-up (Days)	Standard Deviation	Median Follow-up (Days)	Interquartile Range
TAVR Direct Final	27.106	7.511	30	0
BAV TAVR Final	27.945	6.126	30	0

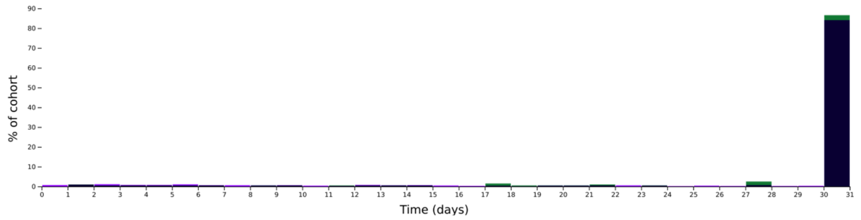

Follow-up Time (After Matching)

Cohort	Mean Follow-up (Days)	Standard Deviation	Median Follow-up (Days)	Interquartile Range
TAVR Direct Final	27.848	6.324	30	0
BAV TAVR Final	27.975	6.128	30	0

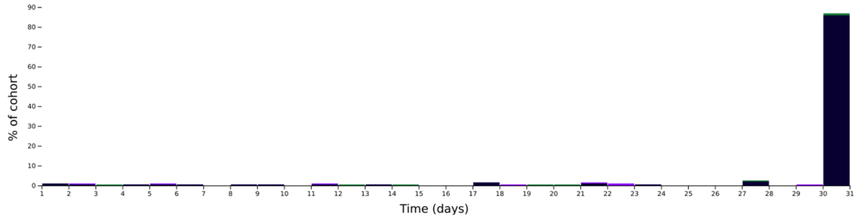

1 Mortality

Risk analysis excluding patients with outcome prior to the time window

Cohort		Patients in cohort	Patients with outcome	Risk
1	TAVR Direct Final	190	29	0.153
2	BAV TAVR Final	193	25	0.130

		95% CI	z	p
**Risk Difference**	0.023	(-0.047, 0.093)	0.649	0.516
**Risk Ratio**	1.178	(0.718, 1.935)	N/A	N/A
**Odds Ratio**	1.210	(0.680, 2.155)	N/A	N/A

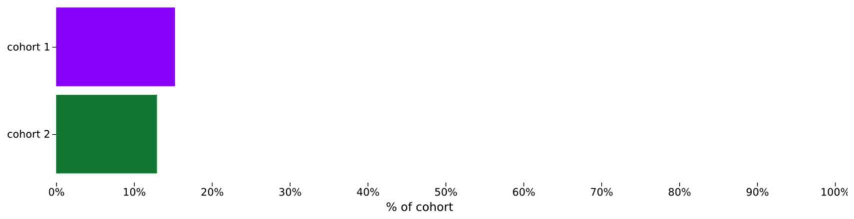

10 patients in Cohort 1 and 10 patients in Cohort 2 were excluded from results because they had the outcome prior to the time window.

Kaplan - Meier survival analysis excluding patients with outcome prior to the time window

Cohort		Patients in cohort	Patients with outcome	Median survival (days)	Survival probability at end of time window
1	TAVR Direct Final	190	29	--	84.45%
2	BAV TAVR Final	193	25	--	86.85%

	χ^2	df	p
**Log-Rank Test**	0.417	1	0.518

	Hazard Ratio	95% CI	χ^2	df	p
**Hazard Ratio and Proportionality**	1.192	(0.698, 2.035)	0.142	1	0.706

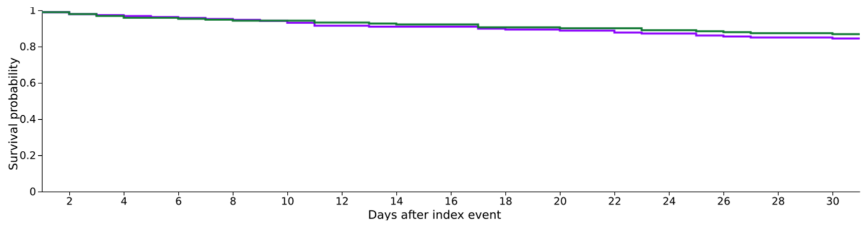

10 patients in Cohort 1 and 10 patients in Cohort 2 were excluded from results because they had the outcome prior to the time window.

2 Pacemaker

Risk analysis excluding patients with outcome prior to the time window

Cohort		Patients in cohort	Patients with outcome	Risk
1	TAVR Direct Final	191	11	0.058
2	BAV TAVR Final	187	13	0.070

		95% CI	z	p
**Risk Difference**	-0.012	(-0.061, 0.037)	-0.475	0.634
**Risk Ratio**	0.828	(0.381, 1.802)	N/A	N/A
**Odds Ratio**	0.818	(0.357, 1.875)	N/A	N/A

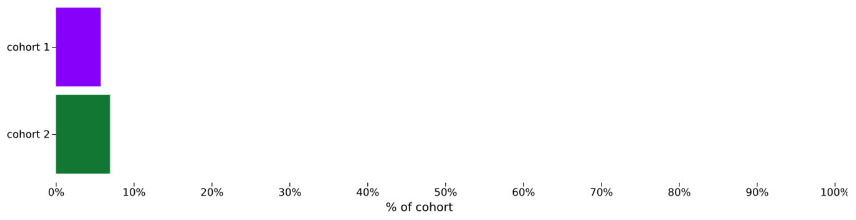

10 patients in Cohort 1 and 11 patients in Cohort 2 were excluded from results because they had the outcome prior to the time window.

Kaplan - Meier survival analysis excluding patients with outcome prior to the time window

Cohort		Patients in cohort	Patients with outcome	Median survival (days)	Survival probability at end of time window
1	TAVR Direct Final	191	11	--	94.12%
2	BAV TAVR Final	187	13	--	92.88%

	χ^2	df	p
**Log-Rank Test**	0.206	1	0.650

	Hazard Ratio	95% CI	χ^2	df	p
**Hazard Ratio and Proportionality**	0.831	(0.372, 1.855)	0.534	1	0.465

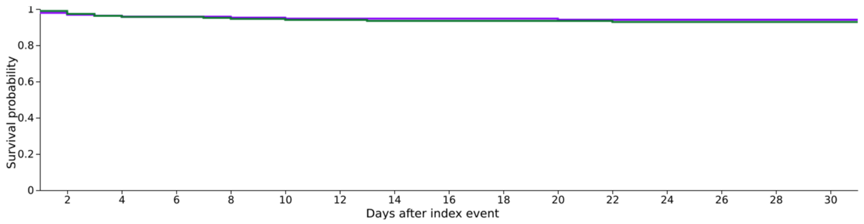

10 patients in Cohort 1 and 11 patients in Cohort 2 were excluded from results because they had the outcome prior to the time window.

3 MACE

Risk analysis

Cohort		Patients in cohort	Patients with outcome	Risk
1	TAVR Direct Final	198	60	0.303
2	BAV TAVR Final	198	54	0.273

		95% CI	z	p
**Risk Difference**	0.030	(-0.059, 0.119)	0.666	0.505
**Risk Ratio**	1.111	(0.815, 1.516)	N/A	N/A
**Odds Ratio**	1.159	(0.750, 1.792)	N/A	N/A

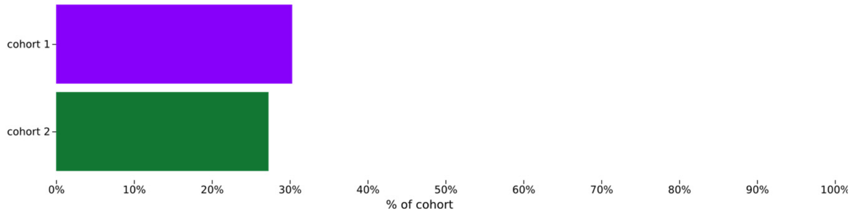

Kaplan - Meier survival analysis

Cohort		Patients in cohort	Patients with outcome	Median survival (days)	Survival probability at end of time window
1	TAVR Direct Final	198	60	--	69.12%
2	BAV TAVR Final	198	54	--	72.27%

	χ^2	df	p
**Log-Rank Test**	0.488	1	0.485

	Hazard Ratio	95% CI	χ^2	df	p

**Hazard Ratio and Proportionality**	1.138	(0.788, 1.643)	0.012	1	0.913

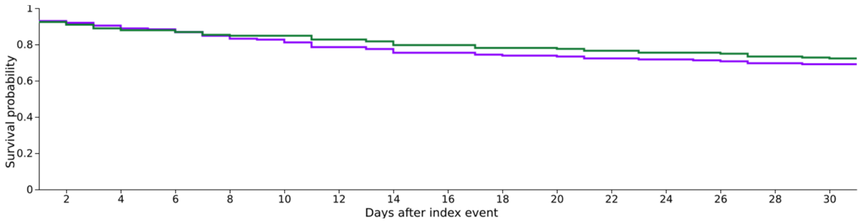

4 Hemodialysis

Risk analysis

Cohort		Patients in cohort	Patients with outcome	Risk
1	TAVR Direct Final	198	14	0.071
2	BAV TAVR Final	198	15	0.076

		95% CI	z	p
**Risk Difference**	-0.005	(-0.056, 0.046)	-0.193	0.847
**Risk Ratio**	0.933	(0.463, 1.882)	N/A	N/A
**Odds Ratio**	0.928	(0.436, 1.978)	N/A	N/A

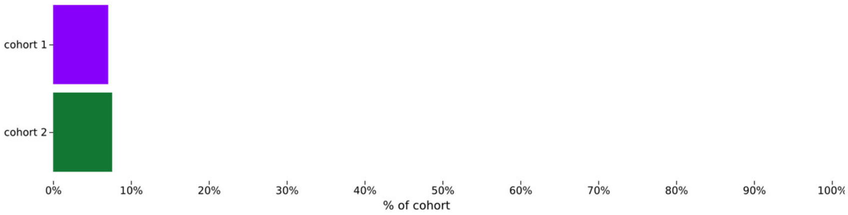

Kaplan - Meier survival analysis

Cohort		Patients in cohort	Patients with outcome	Median survival (days)	Survival probability at end of time window
1	TAVR Direct Final	198	14	--	92.74%
2	BAV TAVR Final	198	15	--	92.28%

	χ^2	df	p
**Log-Rank Test**	0.043	1	0.835

	Hazard Ratio	95% CI	χ^2	df	p
**Hazard Ratio and Proportionality**	0.925	(0.447, 1.917)	0.886	1	0.347

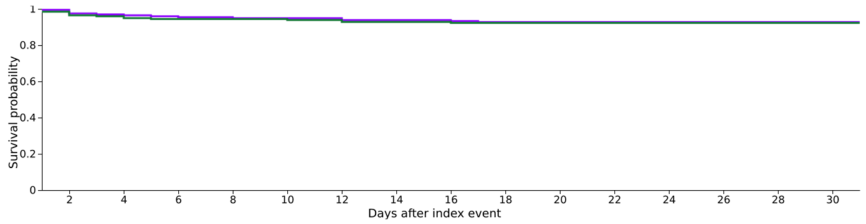

5 Atrial fibrillation

Risk analysis

Cohort		Patients in cohort	Patients with outcome	Risk
1	TAVR Direct Final	198	54	0.273
2	BAV TAVR Final	198	51	0.258

		95% CI	z	p
**Risk Difference**	0.015	(-0.072, 0.102)	0.342	0.733
**Risk Ratio**	1.059	(0.763, 1.470)	N/A	N/A
**Odds Ratio**	1.081	(0.692, 1.689)	N/A	N/A

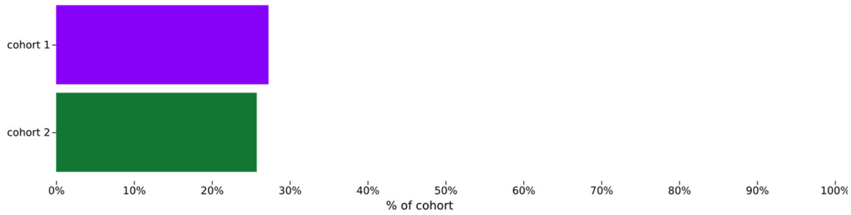

Kaplan - Meier survival analysis

Cohort		Patients in cohort	Patients with outcome	Median survival (days)	Survival probability at end of time window
1	TAVR Direct Final	198	54	--	71.74%
2	BAV TAVR Final	198	51	--	73.05%

	χ^2	df	p
**Log-Rank Test**	0.206	1	0.650

	Hazard Ratio	95% CI	χ^2	df	p
**Hazard Ratio and Proportionality**	1.093	(0.745, 1.603)	2.749	1	0.097

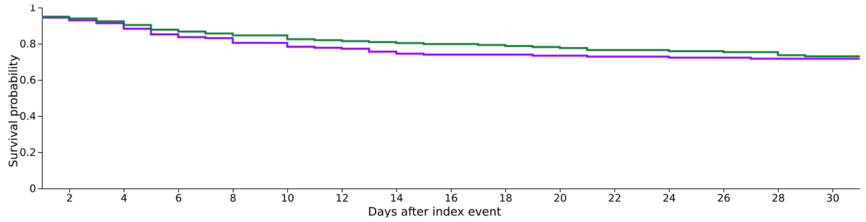

6 HF

Risk analysis

Cohort		Patients in cohort	Patients with outcome	Risk
1	TAVR Direct Final	198	137	0.692
2	BAV TAVR Final	198	139	0.702

		95% CI	z	p
**Risk Difference**	-0.010	(-0.101, 0.080)	-0.219	0.827
**Risk Ratio**	0.986	(0.866, 1.122)	N/A	N/A
**Odds Ratio**	0.953	(0.621, 1.464)	N/A	N/A

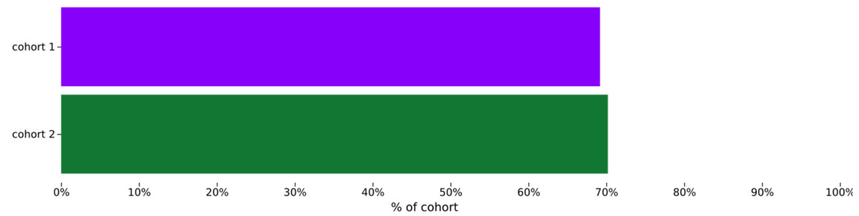

Kaplan - Meier survival analysis

Cohort		Patients in cohort	Patients with outcome	Median survival (days)	Survival probability at end of time window
1	TAVR Direct Final	198	137	7	28.94%
2	BAV TAVR Final	198	139	7	27.78%

	χ^2	df	p
**Log-Rank Test**	0.005	1	0.943

	Hazard Ratio	95% CI	χ^2	df	p
**Hazard Ratio and Proportionality**	1.010	(0.798, 1.279)	0.942	1	0.332

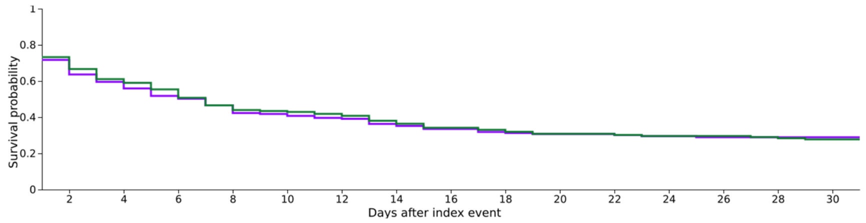

7 AMI

Risk analysis

Cohort		Patients in cohort	Patients with outcome	Risk
1	TAVR Direct Final	198	33	0.167
2	BAV TAVR Final	198	32	0.162

		95% CI	z	p

		95% CI	z	p
**Risk Difference**	0.005	(-0.068, 0.078)	0.136	0.892
**Risk Ratio**	1.031	(0.661, 1.609)	N/A	N/A
**Odds Ratio**	1.037	(0.610, 1.766)	N/A	N/A

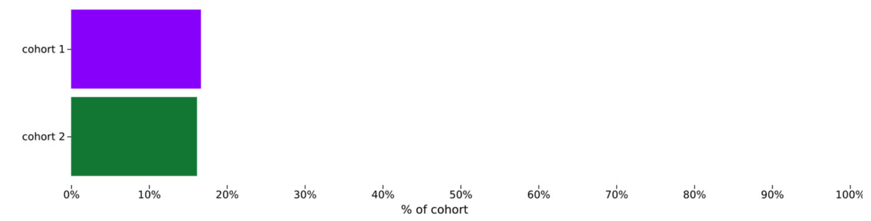

Kaplan - Meier survival analysis

Cohort		Patients in cohort	Patients with outcome	Median survival (days)	Survival probability at end of time window
1	TAVR Direct Final	198	33	--	82.89%
2	BAV TAVR Final	198	32	--	83.39%

	χ^2	df	p
**Log-Rank Test**	0.016	1	0.898

	Hazard Ratio	95% CI	χ^2	df	p
**Hazard Ratio and Proportionality**	1.031	(0.634, 1.677)	0.000	1	0.992

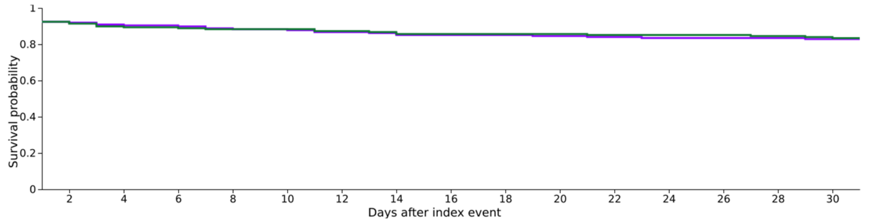

8 Cerebral Infraction

Risk analysis

Cohort		Patients in cohort	Patients with outcome	Risk
1	TAVR Direct Final	198	16	0.081
2	BAV TAVR Final	198	11	0.056

		95% CI	z	p
**Risk Difference**	0.025	(-0.024, 0.075)	0.997	0.319
**Risk Ratio**	1.455	(0.693, 3.055)	N/A	N/A
**Odds Ratio**	1.495	(0.675, 3.307)	N/A	N/A

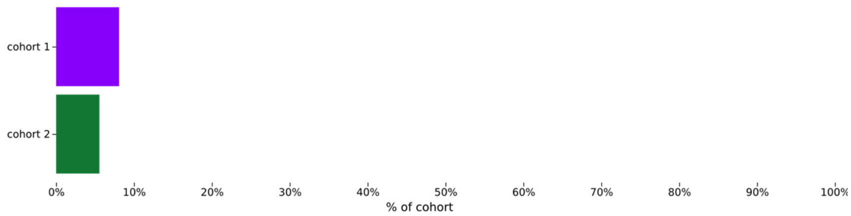

Kaplan - Meier survival analysis

Cohort		Patients in cohort	Patients with outcome	Median survival (days)	Survival probability at end of time window
1	TAVR Direct Final	198	16	--	91.71%
2	BAV TAVR Final	198	11	--	94.28%

	χ^2	df	p
**Log-Rank Test**	1.003	1	0.317

	Hazard Ratio	95% CI	χ^2	df	p
**Hazard Ratio and Proportionality**	1.474	(0.684, 3.176)	0.007	1	0.933

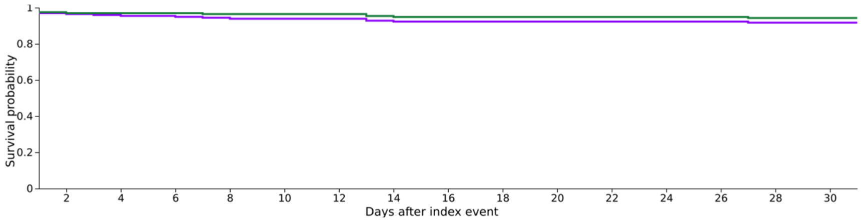

Appendix A – Text Representation of the Cohorts Definition

This section lists all terms used in the definitions of the two cohorts.

Query Criteria for Cohort 1 (query name: TAVR Direct Final)

All the following must be satisfied:

Group 1A: The terms in this group occurred at any time
Patients must have:
all of the following:
Nonrheumatic aortic (valve) stenosis (UMLS:ICD10CM:I35.0); and
Cardiogenic shock (UMLS:ICD10CM:R57.0).
Patients cannot have:
any of the following:
Replacement, aortic valve, open, with cardiopulmonary bypass; with stentless tissue valve (UMLS:CPT:33410); or
Replacement, aortic valve, open, with cardiopulmonary bypass; with prosthetic valve other than homograft or stentless valve (UMLS:CPT:33405); or
Replacement of Aortic Valve with Synthetic Substitute, Open Approach (UMLS:ICD10PCS:02RF0JZ); or
Replacement, aortic valve, open, with cardiopulmonary bypass; with prosthetic valve other than homograft or stentless valve (UMLS:CPT:33405); or
Replacement, aortic valve; with aortic annulus enlargement, noncoronary sinus (UMLS:CPT:33411); or
Replacement of Aortic Valve with Zooplastic Tissue, Open Approach (UMLS:ICD10PCS:02RF08Z); or
Replacement of Aortic Valve with Nonautologous Tissue Substitute, Open Approach (UMLS:ICD10PCS:02RF0KZ); or
Replacement of Aortic Valve with Autologous Tissue Substitute, Open Approach (UMLS:ICD10PCS:02RF07Z); or
Replacement, aortic valve; by translocation of autologous pulmonary valve with allograft replacement of pulmonary valve (Ross procedure) (UMLS:CPT:33413).
Group 1B: Any instance of Group 1B occurred within 10 days on or after any instance of Group 1A
Patients must have:
any of the following:
Transcatheter aortic valve replacement (TAVR/TAVI) with prosthetic valve; open axillary artery approach (UMLS:CPT:33363); or
Transcatheter aortic valve replacement (TAVR/TAVI) with prosthetic valve; open femoral artery approach (UMLS:CPT:33362); or
Transcatheter aortic valve replacement (TAVR/TAVI) with prosthetic valve; percutaneous femoral artery approach (UMLS:CPT:33361); or
Transcatheter aortic valve replacement (TAVR/TAVI) with prosthetic valve; open iliac artery approach (UMLS:CPT:33364); or
Transcatheter aortic valve implantation (UMLS:SNOMED:773996000); or
Transcatheter aortic valve replacement (TAVR/TAVI) with prosthetic valve (UMLS:CPT:1021150); or
Transcatheter aortic valve replacement (TAVR/TAVI) with prosthetic valve; cardiopulmonary bypass support with open peripheral arterial and venous cannulation (eg, femoral, iliac, axillary vessels) (List separately in addition to code for primary procedure) (UMLS:CPT:33368); or
Transcatheter aortic valve replacement (TAVR/TAVI) with prosthetic valve; cardiopulmonary bypass support with central arterial and venous cannulation (eg, aorta, right atrium, pulmonary artery) (List separately in addition to code for primary procedure) (UMLS:CPT:33369); or
Transcatheter aortic valve replacement (TAVR/TAVI) with prosthetic valve; cardiopulmonary bypass

support with percutaneous peripheral arterial and venous cannulation (eg, femoral vessels) (List separately in addition to code for primary procedure) (UMLS:CPT:33367).

Query Criteria for Cohort 2 (query name: BAV TAVR Final)

All the following must be satisfied:

Group 1A: The terms in this group occurred at any time
Patients must have:
all of the following:
Nonrheumatic aortic (valve) stenosis (UMLS:ICD10CM:I35.0); and
Cardiogenic shock (UMLS:ICD10CM:R57.0); and
any of the following:
Percutaneous balloon valvuloplasty; aortic valve (UMLS:CPT:92986); or
Dilation of Aortic Valve, Percutaneous Approach (UMLS:ICD10PCS:027F3ZZ); or
Percutaneous balloon valvuloplasty (UMLS:CPT:1012998); or
Dilation of Aortic Valve, Percutaneous Endoscopic Approach (UMLS:ICD10PCS:027F4ZZ); or
Percutaneous balloon valvuloplasty; aortic valve (UMLS:CPT:92986); or
Percutaneous balloon valvuloplasty of aortic valve (UMLS:SNOMED:77166000).
Patients cannot have:
any of the following:
Replacement, aortic valve, open, with cardiopulmonary bypass; with stentless tissue valve (UMLS:CPT:33410); or
Replacement, aortic valve, open, with cardiopulmonary bypass; with prosthetic valve other than homograft or stentless valve (UMLS:CPT:33405); or
Replacement, aortic valve, open, with cardiopulmonary bypass (UMLS:CPT:1029693); or
Replacement, aortic valve, open, with cardiopulmonary bypass; with prosthetic valve other than homograft or stentless valve (UMLS:CPT:33405); or
Replacement, aortic valve, open, with cardiopulmonary bypass; with allograft valve (freehand) (UMLS:CPT:33406); or
Replacement, aortic valve, open, with cardiopulmonary bypass; with stentless tissue valve (UMLS:CPT:33410); or
Replacement of Aortic Valve with Zooplastic Tissue, Open Approach (UMLS:ICD10PCS:02RF08Z); or
Replacement of Aortic Valve with Synthetic Substitute, Open Approach (UMLS:ICD10PCS:02RF0JZ); or
Replacement of Aortic Valve with Nonautologous Tissue Substitute, Open Approach (UMLS:ICD10PCS:02RF0KZ); or
Replacement of Aortic Valve with Autologous Tissue Substitute, Open Approach (UMLS:ICD10PCS:02RF07Z); or
Replacement of Aortic Valve with Zooplastic Tissue, using Rapid Deployment Technique, Open Approach (UMLS:ICD10PCS:02RF08N); or
Replacement of Aortic Valve using Zooplastic Tissue, Rapid Deployment Technique, Open Approach, New Technology Group 2 (deprecated 2022) (UMLS:ICD10PCS:X2RF032).
Group 1B: Any instance of Group 1B occurred within 1 day and 1 month after any instance of Group 1A
Patients must have:
any of the following:
Transcatheter aortic valve replacement (TAVR/TAVI) with prosthetic valve; open axillary artery approach (UMLS:CPT:33363); or
Transcatheter aortic valve replacement (TAVR/TAVI) with prosthetic valve; open femoral artery approach (UMLS:CPT:33362); or
Transcatheter aortic valve replacement (TAVR/TAVI) with prosthetic valve; transapical exposure (eg,

left thoracotomy) (UMLS:CPT:33366); or
Transcatheter aortic valve replacement (TAVR/TAVI) with prosthetic valve; percutaneous femoral artery approach (UMLS:CPT:33361); or
Transcatheter aortic valve replacement (TAVR/TAVI) with prosthetic valve; open iliac artery approach (UMLS:CPT:33364); or
Transcatheter aortic valve replacement (TAVR/TAVI) with prosthetic valve; transaortic approach (eg, median sternotomy, mediastinotomy) (UMLS:CPT:33365); or
Transcatheter aortic valve implantation (UMLS:SNOMED:773996000); or
Transcatheter aortic valve replacement (deprecated 2022) (UMLS:SNOMED:725351001); or
Transcatheter aortic valve replacement (TAVR/TAVI) with prosthetic valve (UMLS:CPT:1021150); or
Transcatheter aortic valve replacement (TAVR/TAVI) with prosthetic valve; cardiopulmonary bypass support with open peripheral arterial and venous cannulation (eg, femoral, iliac, axillary vessels) (List separately in addition to code for primary procedure) (UMLS:CPT:33368); or
Transcatheter aortic valve replacement (TAVR/TAVI) with prosthetic valve; cardiopulmonary bypass support with central arterial and venous cannulation (eg, aorta, right atrium, pulmonary artery) (List separately in addition to code for primary procedure) (UMLS:CPT:33369); or
Transcatheter aortic valve replacement (TAVR/TAVI) with prosthetic valve; cardiopulmonary bypass support with percutaneous peripheral arterial and venous cannulation (eg, femoral vessels) (List separately in addition to code for primary procedure) (UMLS:CPT:33367).

Appendix B – Text Representation of the Analysis Setup

This section contains the Index Event definition for each cohort.

The index event for Cohort 1 (query name: TAVR Direct Final) is defined as the following:

All the following must be satisfied:

Group 1A: The terms in this group occurred at any time
Patients must have:
all of the following:
Nonrheumatic aortic (valve) stenosis (UMLS:ICD10CM:I35.0); and
Cardiogenic shock (UMLS:ICD10CM:R57.0).
Patients cannot have:
any of the following:
Replacement, aortic valve, open, with cardiopulmonary bypass; with stentless tissue valve (UMLS:CPT:33410); or
Replacement, aortic valve, open, with cardiopulmonary bypass; with prosthetic valve other than homograft or stentless valve (UMLS:CPT:33405); or
Replacement of Aortic Valve with Synthetic Substitute, Open Approach (UMLS:ICD10PCS:02RF0JZ); or
Replacement, aortic valve, open, with cardiopulmonary bypass; with prosthetic valve other than homograft or stentless valve (UMLS:CPT:33405); or
Replacement, aortic valve; with aortic annulus enlargement, noncoronary sinus (UMLS:CPT:33411); or
Replacement of Aortic Valve with Zooplastic Tissue, Open Approach (UMLS:ICD10PCS:02RF08Z); or
Replacement of Aortic Valve with Nonautologous Tissue Substitute, Open Approach (UMLS:ICD10PCS:02RF0KZ); or
Replacement of Aortic Valve with Autologous Tissue Substitute, Open Approach (UMLS:ICD10PCS:02RF07Z); or
Replacement, aortic valve; by translocation of autologous pulmonary valve with allograft replacement of pulmonary valve (Ross procedure) (UMLS:CPT:33413).

Group 1B: Any instance of Group 1B occurred within 10 days on or after any instance of Group 1A
Patients must have:
any of the following:
Transcatheter aortic valve replacement (TAVR/TAVI) with prosthetic valve; open axillary artery approach (UMLS:CPT:33363); or
Transcatheter aortic valve replacement (TAVR/TAVI) with prosthetic valve; open femoral artery approach (UMLS:CPT:33362); or
Transcatheter aortic valve replacement (TAVR/TAVI) with prosthetic valve; percutaneous femoral artery approach (UMLS:CPT:33361); or
Transcatheter aortic valve replacement (TAVR/TAVI) with prosthetic valve; open iliac artery approach (UMLS:CPT:33364); or
Transcatheter aortic valve implantation (UMLS:SNOMED:773996000); or
Transcatheter aortic valve replacement (TAVR/TAVI) with prosthetic valve (UMLS:CPT:1021150); or
Transcatheter aortic valve replacement (TAVR/TAVI) with prosthetic valve; cardiopulmonary bypass support with open peripheral arterial and venous cannulation (eg, femoral, iliac, axillary vessels) (List separately in addition to code for primary procedure) (UMLS:CPT:33368); or
Transcatheter aortic valve replacement (TAVR/TAVI) with prosthetic valve; cardiopulmonary bypass support with central arterial and venous cannulation (eg, aorta, right atrium, pulmonary artery) (List separately in addition to code for primary procedure) (UMLS:CPT:33369); or
Transcatheter aortic valve replacement (TAVR/TAVI) with prosthetic valve; cardiopulmonary bypass support with percutaneous peripheral arterial and venous cannulation (eg, femoral vessels) (List separately in addition to code for primary procedure) (UMLS:CPT:33367).

The index event for Cohort 2 (query name: BAV TAVR Final) is defined as the following:

All the following must be satisfied:

Group 1A: The terms in this group occurred at any time
Patients must have:
all of the following:
Nonrheumatic aortic (valve) stenosis (UMLS:ICD10CM:I35.0); and
Cardiogenic shock (UMLS:ICD10CM:R57.0); and
any of the following:
Percutaneous balloon valvuloplasty; aortic valve (UMLS:CPT:92986); or
Dilation of Aortic Valve, Percutaneous Approach (UMLS:ICD10PCS:027F3ZZ); or
Percutaneous balloon valvuloplasty (UMLS:CPT:1012998); or
Dilation of Aortic Valve, Percutaneous Endoscopic Approach (UMLS:ICD10PCS:027F4ZZ); or
Percutaneous balloon valvuloplasty; aortic valve (UMLS:CPT:92986); or
Percutaneous balloon valvuloplasty of aortic valve (UMLS:SNOMED:77166000).
Patients cannot have:
any of the following:
Replacement, aortic valve, open, with cardiopulmonary bypass; with stentless tissue valve (UMLS:CPT:33410); or
Replacement, aortic valve, open, with cardiopulmonary bypass; with prosthetic valve other than homograft or stentless valve (UMLS:CPT:33405); or
Replacement, aortic valve, open, with cardiopulmonary bypass (UMLS:CPT:1029693); or
Replacement, aortic valve, open, with cardiopulmonary bypass; with prosthetic valve other than homograft or stentless valve (UMLS:CPT:33405); or
Replacement, aortic valve, open, with cardiopulmonary bypass; with allograft valve (freehand) (UMLS:CPT:33406); or

Replacement, aortic valve, open, with cardiopulmonary bypass; with stentless tissue valve (UMLS:CPT:33410); or
Replacement of Aortic Valve with Zooplastic Tissue, Open Approach (UMLS:ICD10PCS:02RF08Z); or
Replacement of Aortic Valve with Synthetic Substitute, Open Approach (UMLS:ICD10PCS:02RF0JZ); or
Replacement of Aortic Valve with Nonautologous Tissue Substitute, Open Approach (UMLS:ICD10PCS:02RF0KZ); or
Replacement of Aortic Valve with Autologous Tissue Substitute, Open Approach (UMLS:ICD10PCS:02RF07Z); or
Replacement of Aortic Valve with Zooplastic Tissue, using Rapid Deployment Technique, Open Approach (UMLS:ICD10PCS:02RF08N); or
Replacement of Aortic Valve using Zooplastic Tissue, Rapid Deployment Technique, Open Approach, New Technology Group 2 (deprecated 2022) (UMLS:ICD10PCS:X2RF032).
Group 1B: Any instance of Group 1B occurred within 1 day and 1 month after any instance of Group 1A
Patients must have:
any of the following:
Transcatheter aortic valve replacement (TAVR/TAVI) with prosthetic valve; open axillary artery approach (UMLS:CPT:33363); or
Transcatheter aortic valve replacement (TAVR/TAVI) with prosthetic valve; open femoral artery approach (UMLS:CPT:33362); or
Transcatheter aortic valve replacement (TAVR/TAVI) with prosthetic valve; transapical exposure (eg, left thoracotomy) (UMLS:CPT:33366); or
Transcatheter aortic valve replacement (TAVR/TAVI) with prosthetic valve; percutaneous femoral artery approach (UMLS:CPT:33361); or
Transcatheter aortic valve replacement (TAVR/TAVI) with prosthetic valve; open iliac artery approach (UMLS:CPT:33364); or
Transcatheter aortic valve replacement (TAVR/TAVI) with prosthetic valve; transaortic approach (eg, median sternotomy, mediastinotomy) (UMLS:CPT:33365); or
Transcatheter aortic valve implantation (UMLS:SNOMED:773996000); or
Transcatheter aortic valve replacement (deprecated 2022) (UMLS:SNOMED:725351001); or
Transcatheter aortic valve replacement (TAVR/TAVI) with prosthetic valve (UMLS:CPT:1021150); or
Transcatheter aortic valve replacement (TAVR/TAVI) with prosthetic valve; cardiopulmonary bypass support with open peripheral arterial and venous cannulation (eg, femoral, iliac, axillary vessels) (List separately in addition to code for primary procedure) (UMLS:CPT:33368); or
Transcatheter aortic valve replacement (TAVR/TAVI) with prosthetic valve; cardiopulmonary bypass support with central arterial and venous cannulation (eg, aorta, right atrium, pulmonary artery) (List separately in addition to code for primary procedure) (UMLS:CPT:33369); or
Transcatheter aortic valve replacement (TAVR/TAVI) with prosthetic valve; cardiopulmonary bypass support with percutaneous peripheral arterial and venous cannulation (eg, femoral vessels) (List separately in addition to code for primary procedure) (UMLS:CPT:33367).

Appendix C – Text Representation of the Outcomes Definition

This analysis includes the following outcomes:

Mortality
Patients must have:
any of the following:
Deceased (Deceased); or
Ill-defined and unknown cause of mortality (UMLS:ICD10CM:R99); or

Ill-defined and unknown cause of mortality (R99) (UMLS:ICD10CM:R99-R99); or
Illness, unspecified (UMLS:ICD10CM:R69).

Pacemaker
Patients must have:
any of the following:
Insertion of new or replacement of permanent pacemaker with transvenous electrode(s); atrial and ventricular (UMLS:CPT:33208); or
Insertion of new or replacement of permanent pacemaker with transvenous electrode(s); ventricular (UMLS:CPT:33207); or
Transcatheter insertion or replacement of permanent leadless pacemaker, right ventricular, including imaging guidance (eg, fluoroscopy, venous ultrasound, ventriculography, femoral venography) and device evaluation (eg, interrogation or programming), when performed (UMLS:CPT:33274); or
Dual-Chamber Leadless Pacemaker (UMLS:CPT:1037892).

MACE
Patients must have:
any of the following:
Deceased (Deceased); or
ST elevation (STEMI) myocardial infarction of anterior wall (UMLS:ICD10CM:I21.0); or
ST elevation (STEMI) myocardial infarction of inferior wall (UMLS:ICD10CM:I21.1); or
ST elevation (STEMI) myocardial infarction of other sites (UMLS:ICD10CM:I21.2); or
ST elevation (STEMI) myocardial infarction of unspecified site (UMLS:ICD10CM:I21.3); or
Non-ST elevation (NSTEMI) myocardial infarction (UMLS:ICD10CM:I21.4); or
Acute myocardial infarction, unspecified (UMLS:ICD10CM:I21.9); or
Acute myocardial infarction (UMLS:ICD9CM:410); or
Cerebral infarction due to thrombosis of precerebral arteries (UMLS:ICD10CM:I63.0); or
Cerebral infarction due to embolism of precerebral arteries (UMLS:ICD10CM:I63.1); or
Cerebral infarction due to unspecified occlusion or stenosis of precerebral arteries (UMLS:ICD10CM:I63.2); or
Cerebral infarction due to thrombosis of cerebral arteries (UMLS:ICD10CM:I63.3); or
Cerebral infarction due to embolism of cerebral arteries (UMLS:ICD10CM:I63.4); or
Cerebral infarction due to unspecified occlusion or stenosis of cerebral arteries (UMLS:ICD10CM:I63.5); or
Other cerebral infarction (UMLS:ICD10CM:I63.8).

Hemodialysis
Patients must have:
any of the following:
Hemodialysis (UMLS:ICD9CM:39.95); or
Hemodialysis (UMLS:SNOMED:302497006); or
Hemodialysis Procedures (UMLS:CPT:1012752).

Atrial fibrillation
Patients must have:
any of the following:
Paroxysmal atrial fibrillation (UMLS:ICD10CM:I48.0); or
Persistent atrial fibrillation (UMLS:ICD10CM:I48.1).

HF

Patients must have:
any of the following:
Heart failure (UMLS:ICD10CM:I50); or
Left ventricular failure, unspecified (UMLS:ICD10CM:I50.1); or
Systolic (congestive) heart failure (UMLS:ICD10CM:I50.2); or
Diastolic (congestive) heart failure (UMLS:ICD10CM:I50.3); or
Combined systolic (congestive) and diastolic (congestive) heart failure (UMLS:ICD10CM:I50.4); or
Other heart failure (UMLS:ICD10CM:I50.8); or
Heart failure, unspecified (UMLS:ICD10CM:I50.9); or
Heart failure (UMLS:ICD9CM:428).

AMI
Patients must have:
any of the following:
Acute myocardial infarction (UMLS:ICD10CM:I21); or
ST elevation (STEMI) myocardial infarction of anterior wall (UMLS:ICD10CM:I21.0); or
ST elevation (STEMI) myocardial infarction of inferior wall (UMLS:ICD10CM:I21.1); or
ST elevation (STEMI) myocardial infarction of other sites (UMLS:ICD10CM:I21.2); or
ST elevation (STEMI) myocardial infarction of unspecified site (UMLS:ICD10CM:I21.3); or
Non-ST elevation (NSTEMI) myocardial infarction (UMLS:ICD10CM:I21.4); or
Acute myocardial infarction, unspecified (UMLS:ICD10CM:I21.9); or
Acute myocardial infarction (UMLS:ICD9CM:410).

Cerebral Infraction
Patients must have:
any of the following:
Cerebral infarction (UMLS:ICD10CM:I63); or
Cerebral infarction due to thrombosis of precerebral arteries (UMLS:ICD10CM:I63.0); or
Cerebral infarction due to embolism of precerebral arteries (UMLS:ICD10CM:I63.1); or
Cerebral infarction due to unspecified occlusion or stenosis of precerebral arteries (UMLS:ICD10CM:I63.2); or
Cerebral infarction due to thrombosis of cerebral arteries (UMLS:ICD10CM:I63.3); or
Cerebral infarction due to embolism of cerebral arteries (UMLS:ICD10CM:I63.4); or
Cerebral infarction due to unspecified occlusion or stenosis of cerebral arteries (UMLS:ICD10CM:I63.5); or
Cerebral infarction, unspecified (UMLS:ICD10CM:I63.9).
